# Mucosal-Associated Invariant T Cell Features and TCR Repertoire Characteristics During the Course of Multiple Sclerosis

**DOI:** 10.3389/fimmu.2019.02690

**Published:** 2019-11-20

**Authors:** Edgar Carnero Contentti, Mauricio F. Farez, Jorge Correale

**Affiliations:** ^1^Neuroimmunology Unit, Department of Neuroscience, Hospital Alemán, Buenos Aires, Argentina; ^2^Centro para el Estudio de Enfermedades Neuroinmunologicas (CIEN), Fundación para la Lucha contra las Enfermedades Neurológicas de la Infancia (FLENI), Buenos Aires, Argentina; ^3^Department of Neurology, Fundación para la Lucha contra las Enfermedades Neurológicas de la Infancia (FLENI), Buenos Aires, Argentina

**Keywords:** MAIT cells, multiple sclerosis, neuroimmunology, MRI, CSF (cerebrospinal fluid)

## Abstract

**Objective:** To investigate the frequency, phenotype, function, and longitudinal repertoire of mucosal-associated invariant T (MAIT) cells in relapsing remitting multiple sclerosis (RRMS) and primary progressive multiple sclerosis (PPMS) patients.

**Methods:** Forty-five RRMS patients in remission, 20 RRMS patients experiencing exacerbations, 15 PPMS patients, and 30 healthy controls (HCs) were included in the study. MAIT cells were identified phenotypically as CD3^+^ TCRγδ^−^ Vα7.2 + CD161^high^. In 15 patients, MAIT cell number and MRI lesions were evaluated every 6 months, for 36 months. MAIT cell TCRVβ repertoire was defined using single-cell cloning and mRNA sequencing.

**Results:** Circulating MAIT cells were significantly reduced in both RRMS and PPMS patients, particularly during exacerbations, compared to healthy subjects. This decrease was accompanied by pro-inflammatory cytokine production (TNF-α, IFN-γ, IL-17, and GM-CSF). Three months post-exacerbation, peripheral blood MAIT cell percentages increased significantly along with clinical recovery. Likewise, we observed inverse correlation between MRI lesions and peripheral blood MAIT cell numbers. In paired samples, MAIT cell percentage was significantly higher in CSF than in peripheral blood, suggesting MAIT cell migration through the blood–brain barrier. Finally, MAIT cells showed limited TCRVβ repertoires, in both CSF and peripheral blood, which remained stable over time.

**Conclusions:** MAIT cell levels correlated with MS course both clinically and radiologically, showing marked and sustained oligoclonality. These findings may contribute to a better understanding of pathophysiological phenomena underlying the course of MS, and discovery of MAIT cell inhibitors could pave the way for the development of new therapeutic strategies.

## Introduction

Although the etiology of multiple sclerosis (MS) remains elusive, it is now known that environmental factors and susceptible genes are involved in disease pathogenesis, with immunological, genetic, and histopathology studies of MS patients showing that autoimmunity plays a key role (1–3). In addition to adaptive T and B cell immune responses (4), recent studies have identified new populations of non-conventional T cells called invariable natural killer T (iNKT) cells and mucosal-associated invariant T (MAIT) cells, which function at the interface between adaptive immunity and innate immunity, and also play a role in different autoimmune (3–6). In humans, MAIT cells display a restricted αβ T cell receptor (TCR), in which the TCRVα chain comprises a canonical Vα7.2-Jα33 (from now on the IMGT denomination will be used: TRAV1-2-TRAJ33) rearrangement, paired with a limited number of TCRβ chains: TRBV20-1, TRBV6-1, TRBV6-4, TRBV6-5, and TRBV-13 (7, 8), and less frequent usage of the non-canonical TRAV1-2-TRAJ12/20 TCR rearrangement (8, 9). They can be identified by staining for TRAV1-2 and either CD161, or IL-18Rα, in the TCRγδ^−^CD4^−^CD3^+^ compartment (10). MAIT cells are restricted by MR1, a non-polymorphic class Ib-related MHC molecule (11), and recognize a limited antigen repertoire, represented by molecules that are precursors or derived from riboflavin and the metabolism of folic acid in bacteria and yeast (12, 13). Until now, no autoantigen recognized by MAIT cells has been reported. However, recent studies indicate that in addition to MAIT cells that recognize riboflavin- and folate-related metabolites, human blood contains MR1-restricted T cells, which recognize cell-derived antigens, participating in different inflammatory mechanisms involving B cells and dendritic cells. (14). These data expand the function of MR1 beyond the presentation of microbial antigens, indicating that MR1-restricted cells can participate in the development of different inflammatory processes.

MAIT cells are abundant in tissues exposed to microbial antigens, including lamina propria of the gut and liver, important sites of host–environment interactions. They can be activated in an MR1-dependent or -independent manner. After activation, MAIT cells proliferate, secreting pro-inflammatory cytokines including IFN-γ, TNF-α, GM-CSF, and IL-17, as well as cytotoxic effector molecules such as perforin and granzymes (15–18), resulting in lysis of infected cells (19). These properties suggest that MAIT cells may function as first responders to aberrant microbial signals, while also sustaining abnormal inflammatory reactions (19).

In adult humans, MAIT cells represent up to 10% of circulating T cells, 20–45% of T cells in the liver, and 3–5% of lymphoid cells in the intestinal mucosal (20, 21). Currently, although their preferential distribution at various mucosal sites indicates a role in host responses at the site of pathogen entry, little is known about the roles of these cells in immune-mediated diseases, such as MS (10). MAIT cells have been found in CNS lesions from MS patients (22, 23), which raises questions on their function within the CNS and their relevance in disease pathogenesis. Contradictory observations have been made in different MS cohorts, reporting either a protective or deleterious effect of MAIT cells as well as in experimental autoimmune encephalomyelitis (EAE) (23–28).

In the present study, we investigated frequency, phenotype, function, and longitudinal repertoire of MAIT cells in relapsing remitting and primary progressive MS patients. The multifaceted nature of MAIT cells makes them promising candidates both for therapeutic targeting and as potential biomarkers of disease.

## Methods

### Study Design and Patient Selection

Eighty-six patients diagnosed with clinically definitive MS were recruited through the MS Clinic at the Raúl Carrea Institute for Neurological Research, FLENI. MS was diagnosed according to 2010 revised McDonald criteria. Patients were divided into three groups: (i) relapsing remitting MS in remission (RRMS; *n* = 46), (ii) relapsing remitting MS experiencing acute exacerbations (RREMS; *n* = 25), and (iii) primary progressive MS cases (PPMS; *n* = 15). Exacerbations were defined as development of new symptoms, or worsening of pre-existing ones, confirmed on neurological examination and lasting at least 24 h, in the absence of fever, preceded by stability or improvement lasting at least 30 days. MS patients who were clinically stable for 6 months prior to enrollment or more, and who did not present new T2 or Gd-enhancing lesions on magnetic resonance imaging (MRI), were considered to be in remission. No patients had received steroids or immunosuppressant treatment for at least 6 months prior to study entry. Thirty-nine patients (85%) in remission, and 20 (82%) experiencing exacerbation were on immunomodulatory treatment (interferon β 1a) at study entry. The remaining RRMS (*n* = 7) and RREMS (*n* = 5) patients did not receive immunomodulatory or immunosuppressive treatment. None of the PPMS patients received specific immunosuppressive treatment.

Thirty healthy age- and gender-matched individuals served as controls (HCs). Underlying conditions were ruled after thorough clinical and neurological examination, as well as standard blood biochemistry tests.

Fifteen relapsing remitting MS patients presenting acute exacerbations (10 women and 5 men; mean age 34.8 ± 6.3) were followed for 37.3 ± 3.3 months. Every 3 months, patients underwent complete physical exam including disease activity and Expanded Disability Status Scale Score (EDSS) assessment. Brain MRI was performed at 6-months intervals on a 1.5-T Signa unit (General Electric) and axial slices, 5 mm thick, were obtained with T2-weighted, proton density, fast spin echo, fluid-attenuated inversion recovery, and T1-weighted sequences, before and after administration of gadolinium (Gd) diethylenetriamine penta-acetic acid 0.1 mmol/kg. Results are reported as combined unique activity (CUA), calculated by combining new or enlarging T2 lesions with gadolinium-enhancing lesions as previously reported elsewhere (29).

Heparinized peripheral blood samples were drawn at time of MRI to study MAIT cells. During relapses, peripheral blood was drawn within 3–5 days of the onset of the symptoms. When peripheral blood was drawn from patients with PPMS, no contrast-enhanced lesions were evidenced in any of them. Therefore, the presence of radiological exacerbations could not be considered.

Patients who experienced exacerbation were treated with methylprednisolone 1 g/day for 5 days. In these patients, peripheral blood samples were obtained before steroids treatment. Paired samples [blood and cerebrospinal fluid (CSF)] were obtained in 12 patients during exacerbations (eight women and four men; mean age 35.7 ± 7.1 years). CSF samples were obtained only as part of diagnostic screening in all patients. CSF samples from 12 individuals (eight women and four men; mean age 32.5 ± 6.9 years) undergoing extraspinal orthopedic surgery, obtained prior to spinal anesthetic injection, served as controls. Demographic and clinical characteristics of patients and controls are summarized in Table 1.

**Table 1:** Demographic and clinical characteristics of the study population^a^.

**Characteristics**	**RRRMS**	**RREMS**	**PPMS**	**Controls**
*n*	46	25	15	30
Age (years)	33.5 ± 6.8	35.3 ± 7.1	47.5 ± 3.5	37.3 ± 8.9
Female:male	30:16	16:8	8:7	20:10
EDSS	2.1 ±1.7	2.3 ± 1.8	4.6 ± 2.4	—
Disease duration (years)	3.8 ± 4.3	4.1 ± 5.1	4.3 ± 4.2	—
Patients under IFN-β1a treatment	39 (85%)	20 (82%)	—	—

a *Values are expressed as mean ± SD. IFN, interferon; RRRMS, relapsing remitting MS during remission; RREMS, relapsing remitting MS during exacerbations; PPMS, primary progressive MS; EDSS, Expanded Disability Status Scale Score*.

The IRB of the Dr. Raúl Carrea Institute for Neurological Research, FLENI, approved this study, and written informed consent was obtained from all participants.

### Flow Cytometry

Peripheral blood mononuclear cells (PBMCs) were isolated by density-gradient centrifugation over Ficoll-PaquePLUS (Amersham Biosciences, Piscataway, NJ), and cell surface molecule labeling was performed, using appropriate monoclonal antibody (mAb) combinations including CD4-V500 (RPA-T4), CD8-V450 (RPA-T8), CCR5-PE (2D7), CD162-PE (KPL-1), CD69-PerCP-Cy5.5 (FN50), CD25-FITC (M-A251), CD58-PE (L306.4), VLA-4-PE (9F10), CD19-PE (HIB19), CD218a-PE (H44), and CD45RA-FITC (L48) from BD Biosciences (San Jose, CA); CD3-Alexa 700 (UCTH), TCR Vα7.2-FITC (3C10), TCR Vα7.2-PE-Cy7 (3C10), CD161-PE (HP-3G10), CD161-APC (HP-3G10), TCR-γ/δ-FITC (B1), and TCR-γ/δ-APC (B1) from Biolegend (San Diego, CA); CCR6-PE (53103) and CXCR6-PE (56811) from R&D Systems (Minneapolis, MN); and CD11a-PE (ab27351) from abcam (Cambridge, MA). Isotype control mAbs were purchased from R&D Systems. Samples were acquired with an LSRII flow cytometer (BD Biosciences) using BD FACSDiva™ software, and analyzed with FlowJo software (Tree Star, Ashland, OR).

The size of a population (for example, MAIT cells) was reported as a percentage relative to the overall number of cells acquired in the sample under study. On the other hand, when the entire populations stain with different levels of antibody, reflecting different levels of expression of certain molecules (e.g., adhesion molecules and chemokine receptors), values were reported as MFI, since the distribution of our populations was not bimodal, and the percentages of the coefficient of variation (CV) were <7% in all cases.

### Identification of MAIT Cells

**Figure 1:**
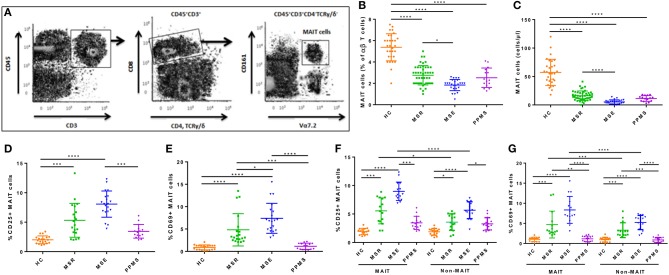
Frequency and absolute number of MAIT cells in peripheral blood. **(A)** Gating strategy to identify CD3^+^CD4^−^TCRγδ^−^CD161^high^TRAV1-2^+^ MAIT cells in peripheral blood from MS patients **(B,C)** Blood MAIT cell frequency from 86 patients with MS (MS remission = 46, MS exacerbations = 25, PPMS = 15) were significantly lower than in 30 HCs, with greater decrease observed during MS exacerbations. **(D)** Frequency of CD25^+^ MAIT cells was significantly higher in all groups of MS patients compared to HCs, with greater increase during MS exacerbations. **(E)** CD69 was upregulated in MAIT cells from RRMS patients (both during remission and exacerbations) compared to HCs, but not in PPMS. **(F,G)** The frequency of CD69^+^ and CD25^+^ cells among MAIT cells was significantly higher than among non-MAIT cells in blood samples from 30 RRMS patients (in remission = 15, during exacerbation= 15), with greater increase observed during exacerbations. CD69 and CD25 expression in both MAIT cells and non-MAIT cells isolated from RRMS patients, was higher than in HC (*n* = 15) or PPMS patients (*n* = 15). Each symbol represents values from a single individual and the middle line represents the mean. Data are shown as mean ± SEM. Kruskal–Wallis test of one-way ANOVA and *post-hoc* data analysis applying Dunn's multiple comparison test were performed to analyze differences between groups **(B–G)**. Wilcoxon matched-pairs signed rank test was also used for comparisons between MAIT vs. non-MAIT cell groups **(F, G)**. ^*^p < 0.05, ^**^p < 0.01, ^***^p < 0.001, ^****^p < 0.0001.

MAIT cells were identified phenotypically as CD3^+^CD4^−^TCRγ/δ^−^TRAV1-2^+^CD161^high^ by flow cytometry as previously described (Figure 1A) (10). To confirm that all MAIT cells contained the appropriate cell rearrangements, TCR genotyping in sorted CD3^+^ TCRγ/δ^−^ TRAV1-2^+^ CD161^high^ cells was performed. TCR α-chain sequences spanning CDR3 region (V-J joining region) were amplified using TRAV1-2 (5′-GTCGGTCTAAAGGGTACAGT-3′) and Cα (5′-TTTAGAGTCTCTCAGCTGGTA-3′) primers. Genotyping of MAIT cells from seven different randomly selected RRMS patients and seven healthy control subjects showed that isolated cells had the TRAV1-2-TRJ33 invariant sequences in the CDR3 region, confirming study cells reported to be MAIT cells were in fact MAIT cells.

For CSF immune phenotyping, 8–10 ml of spinal fluid was obtained by lumbar puncture. Only red blood cell-free samples were studied. Because spinal fluid samples are frequently paucicellular with a rapidly decreasing cell viability, CSF was collected in tubes containing stabilization medium (RPMI-1640, with 25 mM HEPES, 1 mM glutamine, 2% penicillin/streptomycin, 5% heat-inactivated fetal bovine serum, and 2,500 IU heparin) in all cases to prevent cell lysis (30). The maximum time elapsed between sampling and data acquisition in the flow cytometer was 3 h. After collection, cells were concentrated by centrifugation (8 min, 500 *g*). Supernantants were aspirated and cells were resuspended in 100–300 μl of PBS. Next, the samples were incubated for 20′ on ice in the dark with each properly titrated mAb, and after incubation, the cells were washed in PBS. For absolute cell number calculation, 100 μl of PBS and 100 μl of CytoCount counting beads (Dako, Glustrop, Denmark) were added. Data were acquired and analyzed immediately after sample preparation was completed. Absolute cell numbers were calculated using the following formula:

(events in cell subset gate/events in counting bead gate) × (counting beads added per tube/volume of CSF sample) = cells/μl (30).

### Quantification of Secreted Cytokines by MAIT Cells

For *in vitro* studies, MAIT cells (CD3^+^CD4^−^TCRγ/δ^−^TRAV1-2^+^CD161^high^) were isolated from PBMCs using a FACSAria™ III cell sorter (BD Biosciences). Although MR1-antigen tetramer staining has been suggested by some authors as the best method for MAIT cell isolation, other studies have demonstrated that not all MR1-antigen tetramer^+^ cells are MAIT cells (14, 31).

To analyze cytokine production, MAIT cells were cultured in 96-well flat-bottom plates at 1 × 10^5^-3 × 10^4^ cells per well with complete RPMI-1640 media supplemented with 10% heat-inactivated fetal bovine serum (FBS; Life Technologies BRL, Grand Island, NY). For mitogen stimulation, MAIT cells were stimulated with a combination of phorbol 12-myristate 13-acetate (PMA; 100 ng/ml) and ionomycin (1 μg/ml; both from Sigma-Aldrich) for 48 h. IFN-γ, IL-4, IL-17, GM-CSF, and TNF-α secretion was measured by single-cell resolution enzyme-linked immunospot (ELISPOT) assay, as described previously (32), using commercially available ELISPOT kits, following manufacturer instructions. IFN-γ, IL-4, IL-17, and TNF-α ELIPSOT detection kits were purchased from R&D Systems, and the GM-CSF ELISPOT detection kit was purchased from Active Bioscience (Hamburg, Germany). Cytokine secretion was measured during MR1-dependent stimulation, for which HeLa cells (American Type Culture Collections, ATCC, Manassas VA) were transfected with MR1 and untransfected HeLa cells were cultured in Eagle's Minimum Essential Media (Sigma-Aldrich), together with *Escherichia coli* (strain D21; ATCC) fixed in 1% paraformaldehyde for 3 min at infectivity levels increased in multiples of 10 (33). After 3 h, HeLa cells were washed and incubated an additional hour with Eagle's Minimum Essential Media supplemented with 10% FBS and 50 μg/ml gentamicin (Sigma-Aldrich). Infected HeLa cells (1 × 10^5^ per well) used as antigen-presenting cells (APCs) were cultured with isolated MAIT cells (1 × 10^5^ cells per well) for 48 h in complete media supplemented with 10% FBS. Cytokine secretion was measured using ELISPOT. For blocking experiments, anti-MR1 (26.5), anti Class-I HLA-ABC (W6/32), or IgG2 isotype control (all from Biolegend) were added 60 min before stimulation at 10 μg/ml final concentration.

In some experiments, MAIT cell clones were tested for cytokine production 7 to 10 days after their most recent stimulation. Cloned MAIT cells were stimulated as previously described, and after 36 (for IL-4) or 72 h (for the other cytokines), supernatants were harvested and stored at −70°C for later tests. Concentrations of IFN-γ, IL-4, IL-17, GM-CSF, and TNF-α in culture supernatants were determined using commercially available ELISA kits (R&D Systems), following manufacturer instructions.

In all cases, the optimal time courses of each cytokine production by cloned and uncloned MAIT cells were established in preliminary experiments and were defined as the maximum cytokine production obtained after each experimental condition.

### Cloning of MAIT Cells

CD3^+^CD4^−^TCRγ/δ^−^TRAV1-2^+^CD161^high^ cells were sorted from PBMCs as previously described and cloned using limiting dilution (9). Purity of the cell sorting was >96%. Cells were stimulated with phytohemagglutinin (PHA, Sigma-Aldrich; 1 μg/ml), and 100 U/ml recombinant human IL-2 (rhIL-2; R&D Systems), in the presence of 1 × 10^5^ autologous irradiated PBMCs. T cell clones were re-stimulated weekly following the same protocol. To obtain CSF MAIT cell clones, fresh CSF-derived mononuclear cells were isolated after CSF centrifugation, resuspended in complete culture media, and expanded using autologous irradiated PBMCs (5 × 10^4^/well) plus PHA. Cells were re-stimulated with rhIL-2 every 7 days, for 3 weeks. After CSF mononuclear cell expansion, MAIT cells were isolated and cloned by limiting dilution as previously described.

### MAIT Cell Clone TCR Sequencing

TCRα and TCRβ chain high-throughput deep RNA sequencing was performed as previously described (9). Briefly, RNA from MAIT cell clones obtained from peripheral blood and CSF was prepared using TRIZOL (Invitrogen, Carlsbad, CA) following manufacturer instructions, and cDNA was synthesized using an Illumina TruSeq RNA sample preparation kit (San Diego, CA), with multiplexing indexes. Purified cDNA libraries were subjected to an indexed Paired-End sequencing run of 2 × 51 cycles on an Illumina HSeq 2000 platform. For CDR3β region analysis, a multiplex PCR system was used to amplify CDR3β sequences from cDNA samples using 52 forward primers for the Vβ gene segment, and 13 reverse primers for the Jβ segment. Using this method, it is possible to obtain a 60-bp fragment capable of identifying the VDJ region encompassing each unique CDR3β (34). Amplicons were sequenced using the Illumina HiSeq platform. Artificially added sequences (Tag, adaptors), and sequences with low-quality scores were removed using a modified nearest-neighbor algorithm. Remaining sequences were used for assignment of TCRα V, Jα, TCRβ V, D, and Jβ genes, based on definitions provided by the International Immunogenetics Information (IMGT, www.imgt.org) database, and results were analyzed using the ImmunoSEQ analyzer toolset.

### Statistical Analysis

Statistical analyses were performed using SPSS v 22 and Prism 6 (GraphPad) software. Kolmogorov–Smirnov test was used to evaluate distribution of variables and Mann–Whitney or Student's *t*-test was performed to compare continuous data between groups as appropriate. Kruskal–Wallis of one-way ANOVA, a nonparametric test, was used to estimate differences between more than two groups and *post-hoc* analysis was conducted using Dunn's multiple comparison test, when appropriate. Wilcoxon–Mann–Whitney test was used to assess whether the treatment had an impact on MAIT cell percentages during both remission and relapse. Wilcoxon matched-pairs signed-rank test was used for comparison between two paired groups. In addition, Spearman's correlation was used to analyze associations between frequency of MAIT cells and new or enlarging T2 lesions, Gd+ lesions, and CUA on brain MRI. The optimal diagnostic cutpoint between the number of MAIT cells and relapses was estimated using a ROC curve with the nearest method (cutpt command in Stata v12.1). For all tests, *p* < 0.05 were considered statistically significant.

## Results

### MAIT Cells Are Reduced in Peripheral Blood of MS Patients

Percentages and absolute numbers of MAIT cells in peripheral blood samples from the different cohorts were determined by flow cytometry. MAIT cells were defined as CD3^+^CD4^−^TCRγ/δ^−^TRAV1-2^+^CD161^high^ (Figure 1A). Because relapsing remitting and primary progressive patients differ significantly in age (Table 1), two control groups were initially evaluated, one similar in age to relapsing remitting patients (*n* = 20), and another to primary progressive ones (*n* = 10). Because no statistically significant differences were observed in percentages or absolute numbers of MAIT cells between groups (*p* = 0.33 and *p* = 0.45, respectively), they were subsequently pooled into one. As illustrated in Figure 1B, percentage of MAIT cells out of the total TCRαβ T cells was significantly lower in both MS patients during remission and PPMS subjects compared to HCs [2.75 ± 0.90 vs. 5.37 ± 1.26 (*p* < 0.0001), 2.52 ± 0.90 vs. 5.37 ± 1.26 (*p* < 0.0001), respectively]. In addition, MAIT cell number decrease was even more robust during MS exacerbations than during remission (1.84 ± 0.52 vs. 2.75 ± 0.90; *p* < 0.05). No significant differences were observed between MS patients in remission or MS patients during exacerbations vs. PPMS (*p* = 0.99 and *p* = 0.48, respectively). RRMS patients in remission or experiencing exacerbations had significantly lower absolute MAIT cell numbers compared to HCs [16.22 ± 8.38 vs. 57.23 ± 23.05 cells/μl (*p* < 0.0001), and 5.28 ± 3.65 vs. 57.23 ± 23.05 cells/μl (*p* < 0.0001), respectively; Figure 1C]. MAIT cell numbers were also decreased in PPMS patients compared to HCs [11.33 ± 11.05 vs. 57.23 ± 23.05 cells/μl (*p* < 0.0001), Figure 1C]. There were no statistically significant differences between MS patients in remission and PPMS patients (*p* = 0.55). Of note, frequency of TRAV1-2^+^ CD161^low^ and TRAV1-2^+^ CD161^−^ or TRAV1-2^−^ CD161^high^ cell populations did not differ between MS patients and HCs, suggesting that reduced MAIT cell numbers in MS patients were not due to down-modulation of TRAV1-2^+^ TCR or CD161 molecules in MAIT cells of MS patients.

Human MAIT cells predominantly express the CD8α co-receptor (CD8^+^), with a smaller subset not expressing any CD4^−^ CD8^−^ (double negative; DN), and very few CD4^+^ cells expressing the canonical TCR. Recently, significant functional differences have been described between both MAIT cell subsets, with important implications during infectious and inflammatory diseases. The findings suggest that the DN MAIT cell subset may derive from the main CD8^+^ MAIT cell subpopulation (35). We therefore decided to investigate the frequency and number of both subsets in the different study populations. As illustrated in Supplementary Figures S1A,B, both the percentage and absolute number of total MAIT cells and CD8^+^ MAIT cells were significantly reduced in peripheral blood samples from RRMS patients in remission and during exacerbations, as well as in PPMS subjects compared to HC (*p* < 0.0001). Likewise, both percentage and absolute number of DN MAIT cells were also lower in all three MS patient cohorts compared to HC, although the drop was of lesser magnitude (*p* < 0.01 to *p* < 0.001).

Even though the number of untreated patients was small, we found no statistically significant differences in the percentage of MAIT cells between untreated [median, range (2.5; 1–5)] and IFN-β 1a-treated (3; 2–4.5) remission patients (*p* = 0.69). Likewise, there were no differences in untreated (1.95; 0.5–2.6) and IFN-β 1a-treated (2; 1.8–2.5) relapsing patients (*p* = 0.58). These data suggest that the decrease in MAIT cells is part of the pathophysiological process of the disease and not a consequence of the treatment received. On the other hand, since none of the PPMS patients received specific treatment for MS, we can exclude an effect of specific therapies for MS on the results observed in this group of patients.

### MAIT Cell Characterization

To assess whether *ex vivo* MAIT cells from MS patients have a different state of activation than those from HCs, we investigated CD25 and CD69 expression. Percentages of CD25^+^ MAIT cell numbers were significantly higher in MS patients during exacerbations than in MS patients in remission or in PPMS patients [8.07 ± 2.23 vs. 5.32 ± 2.87 cells/μl (*p* < 0.0001), and 8.07 ± 2.23 vs. 3.44 ± 1.55 (*p* < 0.0001), respectively; Figure 1D]. CD69 was upregulated in MAIT cells from RRMS patients (both in remission and during exacerbations) compared with HCs [4.82 ± 3.61 vs. 0.89 ± 0.54 (*p* < 0.0001), and 7.39 ± 3.34 vs. 0.89 ± 0.54 (*p* < 0.0001), respectively; Figure 1D], but not in PPMS patients [1.14 ± 0.69 vs. 0.89 ± 0.54 (*p* = 0.81); Figure 1E]. No statistically significant differences were observed in CD69-expressing MAIT cell numbers between RRMS patients during exacerbations vs. remission (*p* = 0.13). Likewise, CD25 and CD69 expression, measured as mean fluorescence intensity (MFI), was significantly higher in RRMS patients than in HC and PPMS, particularly during exacerbations (*p* < 0.05 and *p* < 0.01, respectively). These findings prompted us to investigate whether these observations were MAIT cell-specific or shared by non-MAIT memory cells (defined as CD3^+^CD45RA^−^TRAV1-2^+/−^CD161^low^CD8^+^). In RRMS patients, the frequency of CD69^+^ and CD25^+^ cells was higher among MAIT cells than non-MAIT cells (*p* < 0.05 to *p* < 0.0001; Figures 1F,G). In addition, expression of both activation markers was even more robust during MS exacerbations (*p* < 0.0001). Of note, the percentage of both CD69^+^ and CD25^+^ cells among MAIT cells and non-MAIT cells isolated from RRMS patients in remission and during exacerbations were significantly higher than in HC and PPMS patients (*p* < 0.05 to *p* < 0.0001; Figures 1F,G). Overall, these results indicate that MAIT cell number decrease in MS patients is accompanied by activated phenotype, suggesting that abnormal MAIT cell activation is present in MS patients, particularly during active disease.

**Figure 2:**
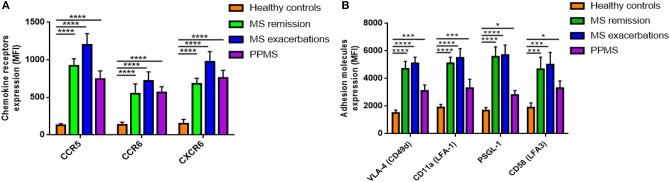
Expression of chemokine receptors and adhesion molecules. **(A)** Expression of CCR5, CCR6, and CXCR6, and **(B)** the adhesion molecules VLA-4 (CD49d), LFA-1 (CD11a), PSGL-1 (CD162), and LFA-3 (CD58) was measured by flow cytometry and results are expressed as mean fluorescence intensity (MFI). MAIT cell values were significantly higher in MS patients than in HCs. Data are presented as mean values ± SEM from 40 RRRMS patients, 25 RREMS patients, 15 PPMS patients, and 25 healthy control subjects. Statistical analysis was performed using the Wilcoxon matched-pairs signed rank test. ^*^p < 0.05, ^***^p < 0.001, ^****^p < 0.0001.

Since MAIT cells can migrate to inflamed tissues (20), we next measured surface expression on *ex vivo* MAIT cells of selectins and integrins, chemokine receptors important for CNS invasion. CCR5, CCR6, and CXCR6 expression, measured as MFI, was significantly higher in all three MS patient cohorts than in HCs (*p* < 0.0001; Figure 2A). Once again, we set out to investigate whether these observations were MAIT cell-specific, or shared by non-MAIT memory cells, and found that MAIT cells from MS patients expressed CCR5, CCR6, and CXCR6 at higher levels than non-MAIT cells, with greater expression during exacerbations (*p* < 0.05 to *p* < 0.001; Supplementary Figures S2A–C). Likewise, MAIT cells from MS patients showed increased MFI for different adhesion molecules and integrins, especially those involved in the first step of lymphocyte extravasation into CNS, including VLA-4 (CD49d), LFA-1 (CD11a), PSGL-1 (CD162), and LFA-3 (CD58), compared to HCs (*p* < 0.05 to *p* < 0.0001; Figure 2B). Expression levels of the molecules analyzed did not differ between MS patients in remission or during exacerbations (Figure 2B). Next, we investigated surface expression of adhesion molecules and integrins on MAIT cells and non-MAIT cells, in the same cohort in which we had previously quantified chemokine receptor levels. In MS patients, expression of VLA-4 was significantly higher in MAIT cells than in non-MAIT cells, with greatest expression occurring during exacerbations (*p* = 0.01 to *p* < 0.001; Supplementary Figure S2D), whereas no significant difference was observed between cell populations in LFA-1, PSGL-1, and LFA-3 surface expression (*p* = 0.33 to *p* = 0.69; Supplementary Figures S2E–G). These specific phenotypic characteristics on MAIT cells from RRMS patients (namely, higher levels of CCR5, CCR6, CXCR6, and VLA-4 expression) suggest that MAIT cells are more prone to migrate toward the inflamed CNS.

**Figure 3:**
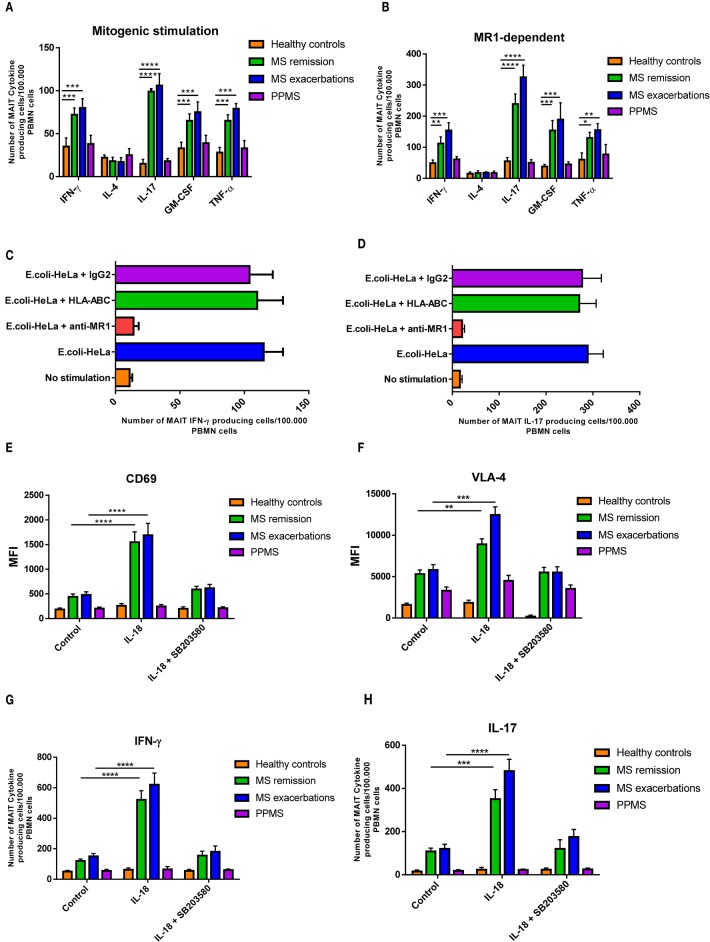
MAIT cells produce pro-inflammatory cytokines after activation. **(A)** For mitogenic stimulation, sorted MAIT cells were stimulated *in vitro* with PMA plus ionomycin, and cytokine secretion was measured using ELISPOT assay. RRMS patients (both in remission and during exacerbation) showed significantly higher numbers of IFN-γ, IL-17, GM-CSF, and TNFα MAIT-producing cells compared to HCs and PPMS patients. In contrast, PPMS and HCs showed similar values of the four cytokines. The number of IL-4 MAIT-producing cells was similar in all four cohorts. **(B)** Similar results were observed when MAIT cells were stimulated in an MR1-dependent manner, using HeLa MR1 transfected cells infected with fixed *E. coli*. Data are presented as mean values ± SEM from 40 RRRMS patients, 25 RREMS patients, 15 PPMS patients, and 25 healthy control subjects. **(C,D)** The response was MAIT cell-specific, since it was blocked by an anti-MR1 monoclonal antibody. Data are presented as mean values ± SEM from 10 RRMS patients in remission. **(E,F)** For TCR-independent activation, *ex vivo* MAIT cells were stimulated with IL-18 (50 ng/ml), in the presence and in the absence of the p38-MAPK inhibitor SB203580 (5 μM; Sigma-Aldrich), and expression of CD69 and VLA-4 was assessed using flow cytometry. Results are presented as mean values ± SEM of mean fluorescence intensity (MFI) from 15 RRMS patients in remission, 15 RRMS patients during exacerbation, 15 PPMS patients, and 15 healthy control subjects. **(G)** Likewise, *ex vivo* isolated MAIT cells were stimulated using a similar protocol and IFN-γ and IL-17 secretion measured using ELISPOT assay. **(H)** Increase in IFN-γ-secreting cell numbers was observed only after adding IL-12 (10 ng/ml) to cultures. Cytokine-secreting cell numbers were calculated by subtracting the number of spots obtained in the absence of stimulation (background control cultures), from the number of spots obtained in cultures exposed to the different stimuli. Data correspond to number of spots per 10^5^ PBMCs. Data are presented as mean values ± SEM from seven different experiments. Statistical analysis was performed using the Wilcoxon matched-pairs signed rank test. ^*^p < 0.05, ^**^p < 0.01, ^***^p < 0.001, ^****^p < 0.0001.

To further explore MAIT function in MS, we analyzed cell cytokine production. To this end, isolated MAIT cells were stimulated with a combination of PMA plus IM. As shown in Figure 3A, RRMS patients generated significantly higher numbers of IFN-γ, IL-17, GM-CSF, and TNFα-producing MAIT cells (*p* < 0.001 to *p* < 0.0001) both during remission and exacerbation than HCs. In contrast, MAIT cell numbers producing any of these cytokines did not differ between PPMS and HCs, while IL-4-producing MAIT cell numbers were similar in all four study cohorts. Similar results were observed when MAIT cells were stimulated in an MR1-dependent manner (*p* < 0.05 to *p* < 0.0001; Figure 3B). To confirm these responses, MAIT cells were isolated *ex vivo* from 10 RRMS patients in remission and were stimulated with HeLa MR1 transfected HeLa cells infected with fixed *E. coli*, in the presence and in the absence of an anti-MR1 blocking antibody. As shown in Figures 3C,D, activation was severely inhibited after addition of the blocking antibody, but not after addition of an MHC Class I blocking antibody (HLA-ABC) or of a control isotype. Taken together, these results suggest that this response is MR1-dependent.

Given the fact that the IL-18 receptor is expressed on MAIT cells (36), and that serum IL-18 is known to be increased in MS patients (23), as well as present in MS CNS lesions (23–37), we set out to investigate whether MAIT cells were activated by IL-18. As shown in Figures 3E–H, MAIT cells isolated *ex vivo* from RRMS patients both in remission and during exacerbations responded robustly to IL-18-driven stimulation with significantly higher expression of CD69, VLA-4, and production of IL-17, compared to PPMS patients and HCs (*p* < 0.01 to *p* < 0.0001). These effects were significantly reduced in the presence of the p38-MAPK inhibitor SB203580, which suppresses IL-18 signaling (38). Overall, these results indicate that MAIT cells can be activated by IL-18 through a TCR-independent mechanism. As previously demonstrated, IFN-γ secretion, however, was not induced by isolated IL-18 stimulation (23), requiring addition of IL-12. Addition of IL-12 to culture medium significantly increased IFN-γ production in RRMS patients both during exacerbation and while in remission, compared to PPMS patients and HC (*p* < 0.0001).

### Variations in MAIT Cell Numbers During MS Course

**Figure 4:**
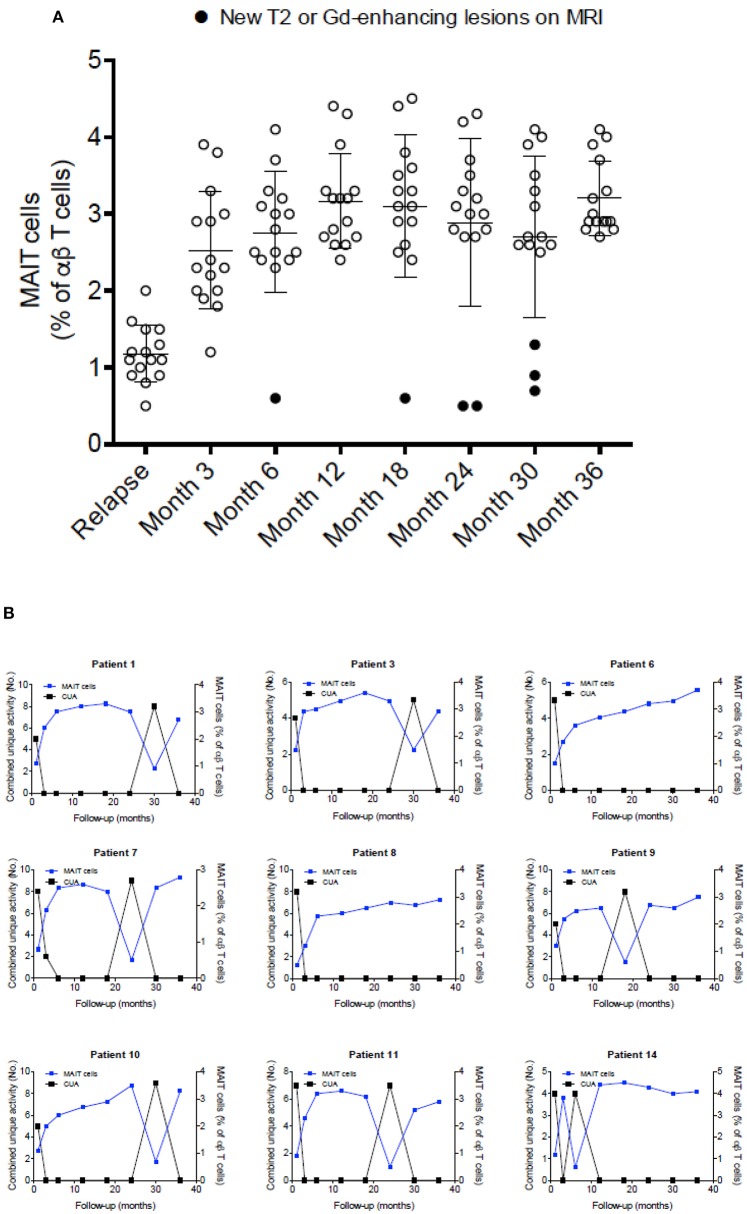
MAIT cell percentages were related to disease activity. **(A)** After a clinical relapse and during follow-up (3–36 months), MAIT cell numbers were significantly increased, in parallel with clinical recovery, with high values persisting while disease remained stable. Each symbol represents values from single individuals (*n* = 15) and the middle line represents the mean. **(B)** During follow-up, seven patients examined at 6, 18, 24, and 30 months showed further decline in MAIT cell numbers (patients numbers 1, 3, 7, 9, 10, 11, and 14), while simultaneously showing disease-related MRI activity (**A**, closed circles), evidenced as new or enlarging T2 lesion and/or Gd-enhancing lesions on brain MRI screening; i.e., inverse correlation was observed between MAIT cell percentage and presence of new or enlarging T2 lesions, Gd-enhancing lesions, or CUA score. In contrast, in MS patients in whom MAIT cell numbers remained elevated during follow-up, there was no evidence of new MRI activity (patients 6 and 8). All patients studied received IFN-β 1a treatment from the beginning of the study was seen. CUA: combined unique activity was calculated by adding new or enlarging T2 lesions to gadolinium-enhancing lesion. Correlations were performed using Spearman's correlation analysis.

**Figure 5:**
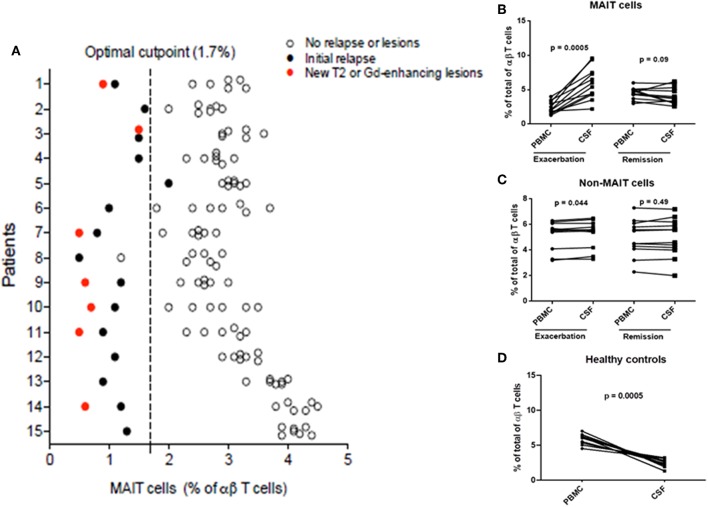
Percentage of MAIT cells pred1icted disease activity. **(A)** Application of a predictive model defining clinical or MRI disease activity as a function of MAIT cell percentage demonstrated a cutoff point of 1.7. **(B)** Decreased circulating MAIT cells in MS were associated with recruitment to the CNS. MAIT cell numbers were significantly higher in CSF than in peripheral blood (paired samples) from 12 RRMS patients during exacerbations (*p* = 0.0005). However, no differences in MAIT cell percentage were found between peripheral blood and CSF in 12 RRMS patients in remission (*p* = 0.09). **(C)** Percentages of non-MAIT cells were also studied in the same group of patients and found to be slightly increased in CSF compared to peripheral blood during MS exacerbations (*p* = 0.044), but not during remissions (*p* = 0.49). **(D)** Conversely, significantly lower numbers of MAIT cells were found in CSF compared to peripheral blood from healthy controls (*p* = 0.0005). For figures **(B–D)**, statistical analysis was performed using the Wilcoxon matched-pairs signed rank test. PBMC: peripheral blood mononuclear cells, CSF: cerebrospinal fluid.

To further explore the link between MAIT cells and clinical characteristics, we analyzed MAIT cell levels in 15 RRMS patients followed for at least 36 months, as well as MRI results at 3- and 6-months intervals, after an exacerbation. As shown in Figure 4A, MAIT cell levels were lower at the time of MS relapse. Three months later, MAIT cell percentages increased significantly along with the clinical recovery, persisting at high values while disease remained stable. Interestingly, seven patients (Figure 4B) showed a further decline in MAIT cell percentages at 6, 18, 24, and 30 months (patient numbers 1, 3, 7, 9, 10, 11, and 14) follow-up, coinciding with new or enlarging T2 lesion and/or Gd enhancing lesions on brain MRI. None of the patients presented clinical relapses during this follow-up period. Thus, an inverse correlation between MAIT cell numbers and either new or enlarging T2 lesions (*r* = −0.69, *p* < 0.0001), Gd-enhancing lesions (*r* = −0.72, *p* < 0.0001), or CUA score (*r* = −0.82, *p* < 0.0001) was observed (Figure 4B and Supplementary Figure S3). In contrast, patients in whom MAIT cell percentage remained elevated during follow-up showed no evidence of MRI activity (patients 6 and 8). These results suggest that peripheral blood MAIT cell levels may reflect MS activity. Application of a predictive model defining disease activity as a function of MAIT cell numbers demonstrated a cutoff point of 1.7, with 0.99 sensitivity, 0.95 specificity, and 0.97 AUC (Figure 5A). To examine whether decrease in circulating MAIT cells in MS might be linked to increased cell recruitment into the CNS, paired samples of peripheral blood and CSF were obtained from 12 RRMS patients during exacerbations, 12 RRMS patients in remission, and 12 healthy controls. MAIT cell numbers were calculated using flow cytometry. As illustrated in Figure 5B, MAIT cell frequency was significantly higher in CSF of RRMS patients during exacerbations than in peripheral blood (mean frequency 5.8 ± 0.6 vs. 2.3 ± 0.2%; *p* = 0.0005). By contrast, no difference was observed in MAIT cell percentages between peripheral blood and CSF during remission (mean frequency 4.4 ± 0.2% vs. 3.9 ± 0.3%; *p* = 0.09; Figure 5B). MAIT cell frequency in CSF from healthy controls was statistically significantly lower compared to MS patients during remission (*p* = 0.01) and exacerbation (*p* = 0.0005). Of note, non-MAIT cells were also found to be slightly increased in CSF compared to peripheral blood during MS exacerbations, but not during remissions (*p* = 0.44, and *p* = 0.49, respectively; Figure 5C). Further analysis of HC revealed higher numbers of MAIT cells in peripheral blood compared to CSF (mean frequency 5.8 ± 0.2% vs. 2.1 ± 0.2%; *p* = 0.0005; Figure 5D). Taken together, these observations are consistent with a preferential recruitment of MAIT cells into the CNS during acute exacerbations.

### MAIT Cell T-Cell Receptor Repertoire

Fifteen MS patients were followed over time for a period of at least 3 years. In this cohort, we were able to isolate 34 MAIT cell clones from peripheral blood in seven patients at study entry. In four of these patients, CSF-sorted MAIT cells (yield = 0.5–0.9 cells/μl) were expanded as described in *Methods* and then cloned by limiting dilutions. Using this method, a total of 23 MAIT cell clones were isolated from CSF. Ninety percent of peripheral blood MAIT cell clones expressed the canonical α chain TRAV1-2-TRJ33 transcript. However, TCR sequence analysis showed that some MAIT cell clones transcribed a functional TRAV1-2-TRJ12 (6%) or TRAV1-2-TRJ20 (4%) rearrangement.

All clones were CD4^−^ and CD161^+^. Twenty-five clones expressed the CD8 molecule, whereas nine clones were CD4^−^ CD8^−^ (S1 Table).

**Figure 6:**
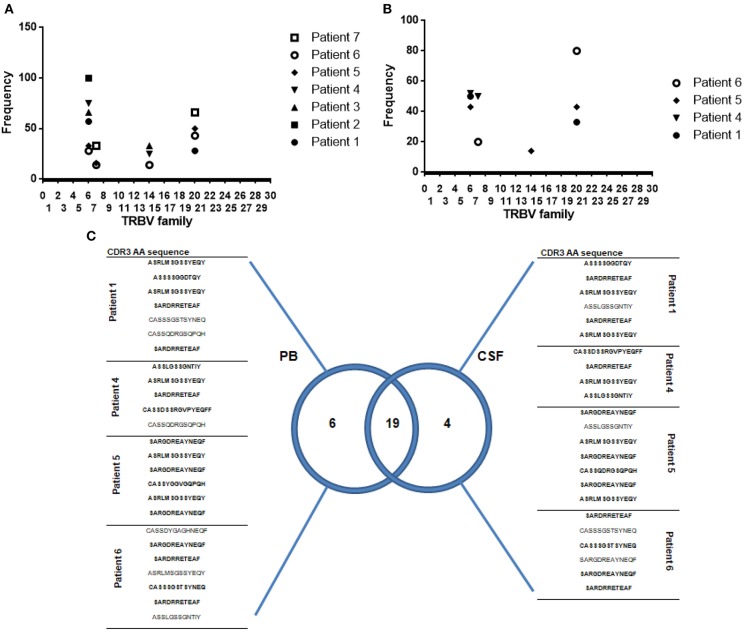
Oligoclonality is a shared feature of both CSF and circulating MAIT cells. **(A,B)** Frequency of Vβ usage by MAIT cell clones (TRAV1-2^+^ CD161^high^) isolated from peripheral blood and CSF. Each symbol represents data from an individual MS patient. **(C)** Venn diagram showing distribution of unique and overlapping TRBV families, selected according to DNA encoding CDR3β amino acid sequences, from peripheral blood and CSF paired samples isolated from four MS patients during relapse. A total of twenty-five MAIT cell clones isolated from peripheral blood and 23 MAIT cell clones isolated from CSF were analyzed. CDR3β amino acid sequences marked in bold represent those common to both PB (peripheral blood) and CSF. TRBV: According to International ImMunoGeneTics information system^®^ standardized nomenclature.

Analysis of TCRVβ sequences in all 34 MAIT cell clones isolated from peripheral blood revealed that they represented a restricted number of CDR3β amino acid clonotypes. As previously reported (7, 9), strong bias of TCRVβ usage was observed: TRBV6-1, TRBV6-4, TRBV6-5, and TRBV20-1 families combined accounted for 79% of total MAIT repertoire (S1 Table and Figure 6A).

To study whether repertoire biases present in peripheral blood of MS patients reflected bias in the CNS, we compared TCRVβ repertoire from paired samples of peripheral blood and CSF. Twenty-three MAIT cell clones isolated from CSF revealed CDR3 clonotype distribution comparable to that observed in peripheral blood MAIT cell clones. TRBV6-1, TRBV6-4, and TRBV20-1 families combined accounted for 78% of the total MAIT repertoire (Figure 6B and S2 Table). These results suggest that oligoclonality is a shared feature of both CSF and circulating MAIT cells (Figure 6C). Furthermore, most MAIT cell clones (82%) expressed high levels of CD103, a defining marker of tissue residency, and on activation, most produced IFN-γ (43%) or IL-17 (39%; S2 Table), suggesting that resident MAIT cells are pro-inflammatory, causing deleterious effects on disease.

To determine whether sorting and cloning strategies used during isolation of CSF and blood MAIT cell clones induced a bias in the repertoire observed, by preferentially expanding certain clonotypes, MAIT cell clones derived from peripheral blood and CSF from 12 healthy controls were also studied. Although the cloning efficiency observed in peripheral blood from RRMS patients and healthy controls was similar, the scarce number of cells present in the CSF of control individuals significantly reduced the cloning efficiency compared to that observed in CSF from RRMS patients (<1% vs. 3–5%). Only in 3 out of 12 HCs were we able to obtain 7 MAIT cell clones. In the same subjects, 16 MAIT cell clones were isolated from peripheral blood. Analysis of CDR3β amino acid sequences in peripheral blood showed a similar repertoire to that of RRMS patients, with TRBV6-1, TRBV6-4, TRBV6-5, and TRBV20-1 families combined accounting for 69% of total MAIT repertoire (S3 Table). Taken together, these findings indicate that no bias was introduced by the cloning strategies used. MAIT cell clones isolated from CSF showed a preferential clonotype distribution represented by TRBV6-1, TRBV6-4, and TRBV6-5, which accounted for 71% of the total MAIT cell repertoire (S3 Table). However, when the oligoclonality observed in peripheral blood and CSF of healthy subjects was analyzed individually, contrary to what was found in RRMS patients, CDR3 clonotypes observed in paired samples of peripheral blood and CSF differed significantly in individual subjects (S3 Table). These results, however, should be interpreted with caution given the low number of MAIT cell clones isolated from CSF in healthy individuals. Next, to explore whether clonotypic characteristics translated into differential functional capacity in MAIT cell clones isolated from peripheral blood and CSF, the clones were stimulated with PMA/ionomycin and subsequent cytokine (IFN-γ, IL-4, IL-17, GM-CSF, and TNF-α) production was assessed. Only in 7 of the 16 MAIT cell clones isolated from peripheral blood was it possible to detect IL-17, IFN-γ, or GM-CSF production. In the case of MAIT cell clones isolated from CSF, only two of the seven clones identified were able to produce IL-17 or IFN-γ (S3 Table). Similar results were observed after *E. coli* stimulation.

**Figure 7:**
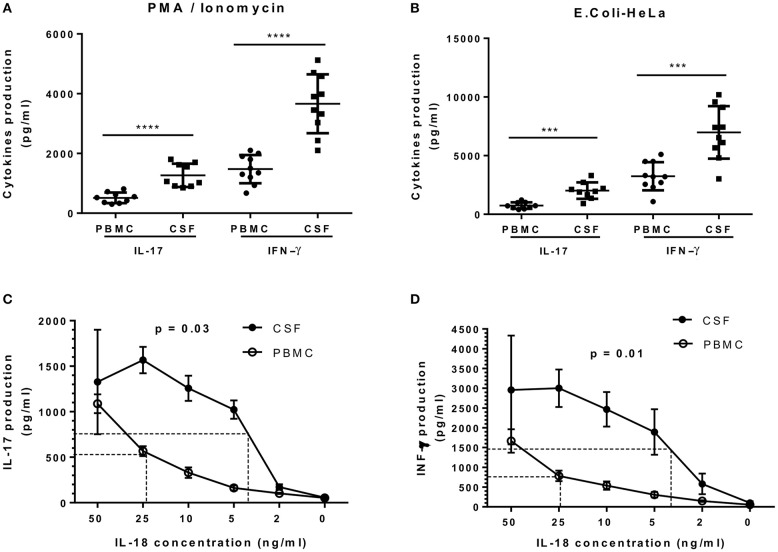
IL-17 and IFN-γ secretion by CSF MAIT cell clones is significantly higher compared to that of peripheral blood MAIT cell clones. **(A)** CSF (*n* = 19) and peripheral blood (*n* = 19) MAIT cell clones were stimulated with PMA/ionomycin and IL-17 (*n* = 9), after which IFN-γ (*n* = 10) was assessed in the supernatants using ELISA. **(B)** Both groups of MAIT cell clones were stimulated in a second protocol using *E. coli*-infected HeLa cells and cytokines assessed in a similar manner. **(C,D)** IL-18 was titrated in CSF and peripheral blood MAIT cell clones and secretion of IL-17 and IFN-γ was assessed using ELISA. Both graphics show that CSF MAIT cell clones are more sensitive to IL-18 stimulation than MAIT cell clones isolated from peripheral blood. In panel **(D)**, IL-18 was titrated in the presence of IL-12 (10 ng/ml). Data represent mean ± SEM of three different MAIT cell clones for each condition. Dotted lines indicate IL-18 concentrations necessary to reach 50% of maximum secretion for each cytokine. Statistical analysis was performed using Mann–Whitney *U* test **(A,B)** and Wilcoxon matched-pairs signed rank test (C, D). ^***^p < 0.001, ^****^p < 0.0001.

Most MAIT cell characterization was first carried out in blood samples. The isolation of several MAIT cell clones from paired samples of CSF and peripheral blood from four RRMS patients provided a unique opportunity to study and compare their functional response. To investigate whether MAIT cell clones isolated from these two different sources presented different functional capacities, each was stimulated with either *E. coli*-infected HeLa cells or with PMA/ionomycin. Production of IL-17 and IFN-γ in the supernatants was then assessed using ELISA. As illustrated in Figure 7A, MAIT cell clones isolated from CSF secreted significantly higher amounts of IL-17 and IFN-γ after PMA/ionomycin stimulation, compared to MAIT cell clones isolated from peripheral blood [520.0 ± 59.4 pg/ml vs. 1060.0 ± 129.9 pg/ml (*p* < 0.0001) and 1425.0 ± 149.8 pg/ml vs. 3675.0 ± 311.2 pg/ml (*p* < 0.0001), respectively]. In a second protocol, MAIT cell clones from both CSF and peripheral blood stimulated with *E. coli*-infected HeLa cells also secreted significantly higher amounts of IL-17 and IFN-γ compared to those from peripheral blood [720.4 ± 92.6 pg/ml vs. 1950.5 ± 234.1 pg/ml (*p* = 0.0003) and 3200.0 ±379.4 pg/ml vs. 6965.0 ± 708.0 pg/ml (*p* < 0.001), respectively; Figure 7B]. Furthermore, stimulation with different concentrations of IL-18 revealed that CSF MAIT cell clones were more sensitive to lower IL-18 concentrations compared to peripheral blood MAIT cell clones in terms of both IL-17 and IFN-γ production (*p* = 0.03 and *p* = 0.01; Figures 7C,D). Indeed, stimulation with IL-18 of CSF MAIT cell clones induced 50% of the maximum secretion of IL-17 at a concentration of 3.5 ng/ml, whereas in peripheral blood MAIT cell clones, a concentration of 23 ng/ml was necessary to achieve a similar effect (6.5 times greater; Figure 7C). Likewise, stimulation of CSF MAIT cell clones with 4 ng/ml of IL-18 induced 50% of the maximum IFN-γ secretion, whereas 25 ng/ml were required in peripheral blood MAIT cell clones (6.25 times more; Figure 7D). Due to the restricted number of cells in CSF, it was not possible to investigate other functional responses. Taken together, these results indicate that CSF MAIT cells respond more strongly in terms of pro-inflammatory cytokine secretion compared to peripheral blood MAIT cells, suggesting that these cells exert a potentially harmful role in MS. Alternatively, the altered cytokine pattern may be due to a different milieu in CSF as compared to peripheral blood.

We next analyzed whether oligoclonality observed in circulating and CSF MAIT cells from RRMS patients remained stable over time. To address this issue, MAIT cell clones were isolated from seven MS patients at 12-months intervals for 3 years, and CDR3β clonotypes were investigated. In every individual, most CDR3β clonotypes identified at study entry were still detectable 36 months later. Some additional clonotypes were detected but not constantly. Results suggest that MAIT cell repertoires in peripheral blood remained stable over the study period (S4 Table).

## Discussion

Current advances in immunology have identified a novel population of innate immune T cells, called MAIT cells (8). Their implication in MS pathogenesis remains elusive, but pro-inflammatory and homing patterns of these cells suggest involvement in lesion-generating processes (10, 22). Decoding whether MAIT cells participate in disease is important to understand MS pathophysiology and eventually develop new therapeutic strategies.

Studies have identified, in mice, clonally expanded T cells expressing the invariant TRAV1-TRJ33 TCR chain, which is highly homologous to the TRAV1-2-TRJ33 TCR chain described in human MAIT cells (7). As MAIT cells in mice are scarce (38), a transgenic (Tg) animal for the MAIT cell-specific TCRα chain (TRAV1-TRJ33) was generated (28). T cells expressing the TRAV1-TRJ33 TCR inhibited induction and progression of EAE, associated with decreased production of Th1 cytokines, and increase of IL-10, occurring at least in part through interaction between B cells and TRAV1-TRJ33 T cells (28).

Reduced numbers of circulating MAIT cells could reflect increased cell death due to sustained activation, or alternatively, greater recruitment toward the inflamed tissues. In the present study, we demonstrated that MAIT cells from MS patients, particularly during exacerbations, expressed high levels of the chemokine receptors CCR5, CCR6, and CXCR6, as well as of the integrin VLA-4. The specific phenotype of MAIT cells from MS patients suggests that they are more prone to migrate to the inflamed CNS. MAIT cell numbers from paired samples of peripheral blood and CSF from RRMS patients were significantly higher in CSF, supporting the theory of MAIT cell migration through the blood–brain barrier (BBB). These findings are consistent with observations in other autoimmune diseases such as type 1 diabetes (T1D), auto-inflammatory intestinal diseases, systemic lupus erythematosus, and rheumatoid arthritis, in which there is depletion of MAIT cells in the periphery and increase in affected tissues (39–42). In agreement with our findings, decreased numbers of circulating MAIT cells and infiltration into the CNS has already been described in RRMS patients by other authors (23, 26). However, in contrast to these results, there have been variable reports on frequencies of MAIT cells in the circulation of RRMS patients compared to controls, with some reporting increased frequencies, no changes in frequencies, or a selective increase in IL-17^+^ CCR6^+^ MAIT cells (25, 27, 43–45). Interestingly, increased IL-17 production by MAIT cells is very likely not just a sign of augmented activation, but rather represents a more pronounced differentiation toward a type-17 phenotype in MS patients, linked to IL-7 signaling (45). Different factors could explain these discrepancies. First, differences in corticosteroid treatment in particular may hamper valid cohort comparisons. In this study, no patients had received steroid or immunosuppressant treatments, for at least 6 months prior to entry. Second, MAIT cell frequency has been shown to decrease with age (46). In our study, no significant differences in age were found between RRMS or PPMS patients and their respective control groups. Therefore, age would not seem to be a determining factor, or explain differences found in peripheral blood MAIT cell numbers between these cohorts. Nevertheless, children with MS showed abnormally increased frequencies and exaggerated pro-inflammatory responses of MAIT cells, which was associated with a relative resistance to suppression by normal Treg cells (43). Third, MAIT cells provoke specific immune responses to distinct microorganisms, in varying magnitude, despite a limited known diversity in riboflavin metabolite antigens (47). The fact that different cohorts come from different countries, exposed to different environmental factors, and especially to varying gut microbiota, could be critical. Finally, methodological differences between studies, particularly differences in gating strategies, could justify the discrepancies observed (27).

Controversy also exists over how MAIT cell recruitment into the CNS through CSF occurs. One early study had shown that although MAIT cells are capable of infiltrating the CNS and expressing VLA-4 (a molecule essential for crossing the BBB), MAIT cells were extremely rare in CSF and present in extremely small numbers compared to those in blood from the same patients (23). The authors suggested that infiltration into the CNS was a consequence of migration through parenchymal vessels and not through the BBB, although they could not entirely rule out CD8^+^ MAIT cell entry through the subarachnoid space. In another study (27), transmigratory capacity of MAIT cells was evaluated through an *in vitro* assay using the human brain endothelial cell line hCMEC/CD3, which mimics BBB features. (48) Clearly, methodological differences between both studies do not allow valid comparisons. The interaction between cell adhesion molecules expressed by endothelial cells of the BBB and their cognate ligands, present on activated leukocytes, plays a central role in transmigration of immune cells into the CNS. In addition, chemokines present on the luminal surface of the endothelium can trigger integrin-dependent adhesion of leukocytes, previously crawling toward interendothelial junctions (49). Similarities between phenotypes of adhesion molecules and chemokines present in conventional T cells and MAIT cells suggest that MAIT cells can migrate into the CNS through a similar route. However, MAIT cells have high expression of adhesion molecules and chemokine receptors even in the remission phase. Nevertheless, the recruitment of MAIT cells to the CNS was different in remission vs. exacerbations. It is possible that, in our study, not all lymphocyte transmigration molecules have been studied, including activated leukocyte cell adhesion molecule (ALCAM), melanoma adhesion molecule (MCAM), and laminin-411. Subpopulations of activated CD4^+^ T cells producing increased levels of IL-17 and GM-CSF preferentially migrate across the BBB compared to cells devoid of these molecules (50–52), suggesting that they facilitate cell entry of pro-inflammatory cytokines producing cells into the CNS during active pathology. Alternatively, MAIT cells during exacerbations can migrate into the CNS by two additional routes: the blood–CSF barrier in the choroid plexus and through meningeal arteries (53). Therefore, more specific studies will be needed to address how MAIT migration patterns are fine-tuned across the BBB.

Decrease in circulating MAIT cell frequency in MS was associated with increased pro-inflammatory cytokine production, particularly IFN-γ, GM-CSF, TNF-α, and IL-17, compared to healthy donors. All these cytokines are considered major players in many autoimmune diseases, including MS. Therefore, contrary to what was observed in mice, infiltrating MAIT cells in MS subjects are probably pro-inflammatory cells causing deleterious effects. Functional differences between TRAV1-Tg mice and human MAIT cells could be explained by differences in MAIT cell development. While Tg mouse MAIT cells displayed a naïve phenotype, MAIT cells in human adults show a memory/effector-like phenotype (54).

Most previous studies on the role of MAIT cells in MS had cross-sectional design, or very short patient follow-up. In the present study, a subgroup of RRMS patients were followed longitudinally for more than 3 years and circulating MAIT cell numbers were linked to clinical and radiological disease activity. Our results show that MS exacerbations were associated with significant reduction in circulating MAIT cells. Interestingly, when blood samples were analyzed 3 months later, circulating MAIT cell numbers were significantly increased in line with clinical recovery, a finding also reflected by dampened MRI disease activity. Decreased circulating MAIT cell numbers observed in some patients during the course disease were also associated with increased MRI activity. Even though the number of MS subjects followed in this study was limited, results nevertheless suggest that MAIT cell levels reflect disease activity, not only playing an active role in MS pathogenesis, but also possibly representing a potential biomarker of disease.

As previously mentioned, MAIT cell traffic between CSF and peripheral blood remains poorly characterized, although it has recently been shown that B-cell clones are shared between CNS, CSF, and peripheral blood in MS patients, with clonal diversification occurring in all three compartments (55). Likewise, CD8 clones identified in brain biopsies have also been found in CSF and blood (56). Our results indicate similar CDR3β chain oligoclonality in MAIT cells from both peripheral blood and CSF from RRMS patients. However, when the oligoclonality observed in peripheral blood and CSF from healthy subjects was analyzed separately, contrary to what was found in RRMS patients, CDR3β clonotypes observed in paired samples of peripheral blood and CSF differed significantly in individual subjects (S3 Table). Furthermore, a greater number of MAIT cell clones capable of producing pro-inflammatory cytokines (IL-17, IFN-γ, and GM-CSF) were identified in both peripheral blood and CSF from patients with RRMS compared to control subjects. In addition, frequency of blood MAIT cells inversely correlated with clinical and MRI disease activity, suggesting that MAIT cells may be involved in MS pathogenesis, and levels found in peripheral blood could reflect inflammatory events within the CNS. Of note, CSF MAIT cell clones produced significantly higher amounts of IL-17 and IFN-γ than peripheral blood MAIT cell clones isolated from the same patients and were more readily stimulated by IL-18, further reinforcing the notion that MAIT cells play a harmful role in MS.

Our results also indicated that peripheral blood MAIT cell repertoires remained stable over time, in agreement with observations from longitudinal studies in which MAIT cells massively expanded in CNS of MS patients at onset, persisted for several years in blood or CSF, and were still being detectable in blood after 18 years (22).

The absence of MAIT cells in the intestinal lamina propria of germ-free mice (11) and their localization under physiological conditions at various mucosal sites, including the intestine, underscores the role of the commensal intestinal microbiota in the expansion of MAIT cells and survival in the periphery. Based on observations from murine models, MAIT cells appear to leave the thymus as näive cells, and then encounter commensal bacterial antigens that drive their maturation, in the periphery (57). This is not however the case in human fetal mucosal tissue of the small intestine, lung, and liver, which have a population of MAIT cells that exhibit a phenotype consistent with mature MAIT cells, with high proliferation capacity. These observations indicate that, in humans, MAIT cells acquire an antimicrobial responsiveness in mucosal tissues before exposure to environmental microbes and the commensal microflora (58). Changes in gut microbiota composition occurring during MS (59) may impact gut permeability, and presence of bacterial ligands may activate MAIT cells, although importance of this interaction needs to be further explored. Alternatively, the presence of endogenous ligands induced during autoinflammatory processes could regulate MAIT cell activation.

Overall, MAIT cells are a relatively new class of unconventional T cell. Their discovery has opened new avenues for the study of autoimmune disease pathogenesis, including that of MS. Correlation with MS activity and alterations prior to T1D onset (60) suggest that MAIT cells could represent a new biomarker for certain autoimmune diseases. Further investigations are warranted on the link between MS, MAIT cell function, and gut microbiota. Finally, discovery of MAIT cell inhibitors (17) may pave the way for the development of new and more promising therapeutic strategies.

## Data Availability Statement

The raw data supporting the conclusions of this manuscript will be made available by the authors, without undue reservation, to any qualified researcher.

## Ethics Statement

This study was reviewed and approved by the local Institutional Ethics Committee (FLENI), and written informed consent was obtained from all participants.

## Author Contributions

JC: conceptualization, funding acquisition, methodology, project administration, resources, supervision, and validation. JC, EC, and MF: data curation, investigation, visualization, and writing—review and editing. JC and EC: writing—original draft.

### Conflict of Interest

EC has received reimbursement for developing educational presentations, educational and research grants as well as professional travel/accommodation stipends from Biogen-Idec, Genzyme, Merck-Serono, Novartis, TEVA Bayer and Roche. MF has received professional travel accommodation stipends from Merck-Serono, TEVA, and Novartis and research funding from Biogen-Idec and Novartis. JC is a board member of Merck-Serono Argentina, Novartis Argentina, Genzyme LATAM, Genzyme global, Biogen-Idec LATAM, and Merck-Serono LATAM. He is on the Steering Committee for the Ofatumumab clinical trials (Novartis Global). JC has received reimbursement for developing educational presentations for Merck-Serono Argentina, Merck-Serono LATAM, Biogen-Idec Argentina, Genzyme Argentina, Novartis Argentina, Novartis LATAM, Novartis Global, and TEVA Argentina as well as professional travel/accommodation stipends.

## Supplementary Material

Supplementary Figure S1 **(A)** Frequency and **(B)** absolute numbers of total, CD8^+^, and DN MAIT cells in peripheral blood, from healthy controls, RRMS patients in remission, RRMS patients during exacerbations, and PPMS patients. Data are presented as mean values ± SEM from seven different experiments. Statistical analysis was performed using the Wilcoxon matched-pairs signed rank test. ^**^*p* < 0.01, ^***^*p* < 0.001, ^****^*p* < 0.0001.

Supplementary Figure S2 **(A–C)** Expression of CCR5, CCR6, and CXCR6 in both MAIT and non-MAIT cells. **(D–G)** Expression of integrins and adhesion molecules VLA-4, LFA-1, PSGL-1, and LFA-3 in both MAIT and non-MAIT cells. In all cases data are presented as mean values ± SEM from 24 RRMS patients (MS in remission = 12 and MS during exacerbations = 12), 12 PPMS patients and 12 healthy controls. Statistical analysis was performed using the Wilcoxon matched-pairs signed rank test **(A–D)**. ^*^*p* < 0.05, ^**^*p* < 0.01, ^***^*p* < 0.001.

Supplementary Figure S3 Correlations between MAIT cell numbers and MRI lesions estimated using Spearman's correlation. **(A)** New Gd+ lesions. **(B)** New or enlarging T2 lesions. **(C)** CUA score.

S1 Table Phenotype and TCRβ amino acid sequences of MAIT cell clones isolated from blood of RRMS patients at the beginning of the study.

S2 Table Phenotype and TCRβ amino acid sequences of MAIT cell clones isolated from CSF of RRMS patients.

S3 Table Phenotype and TCRβ amino acid sequences of MAIT cell clones isolated from peripheral blood and CSF from healthy controls.

S4 Table Longitudinal analysis of the TCRβ chain sequence frequency in clones of MAIT cells isolated from patients with RRMS over time.
