# Supplementary material for: Mucosal-Associated Invariant T Cell Features and TCR Repertoire Characteristics During the Course of Multiple Sclerosis
Source: Front Immunol. 2019 Nov 20;10:2690. doi: 10.3389/fimmu.2019.02690 (PMC6880779; doi:10.3389/fimmu.2019.02690)
Supplement: Supplementary Figure S1 — (A) Frequency and (B) absolute numbers of total, CD8+, and DN MAIT cells in peripheral blood, from healthy controls, RRMS patients in remission, RRMS patients during exacerbations, and PPMS patients. Data are presented as mean values ± SEM from seven different experiments. Statistical analysis was performed using the Wilcoxon matched-pairs signed rank test. **p < 0.01, ***p < 0.001, ****p < 0.0001. [file Image_1.pdf]

Supplementary figure S1

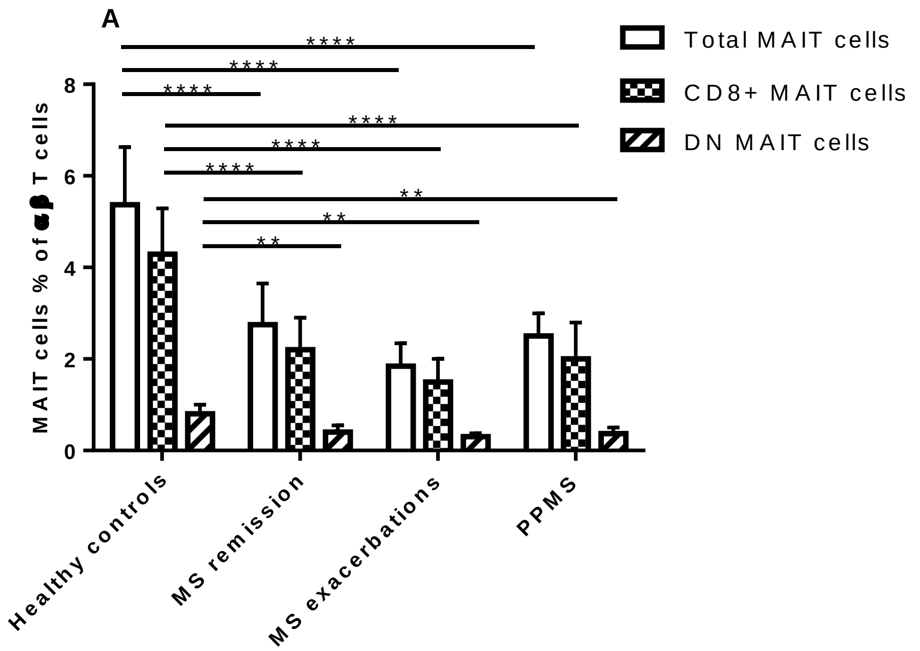

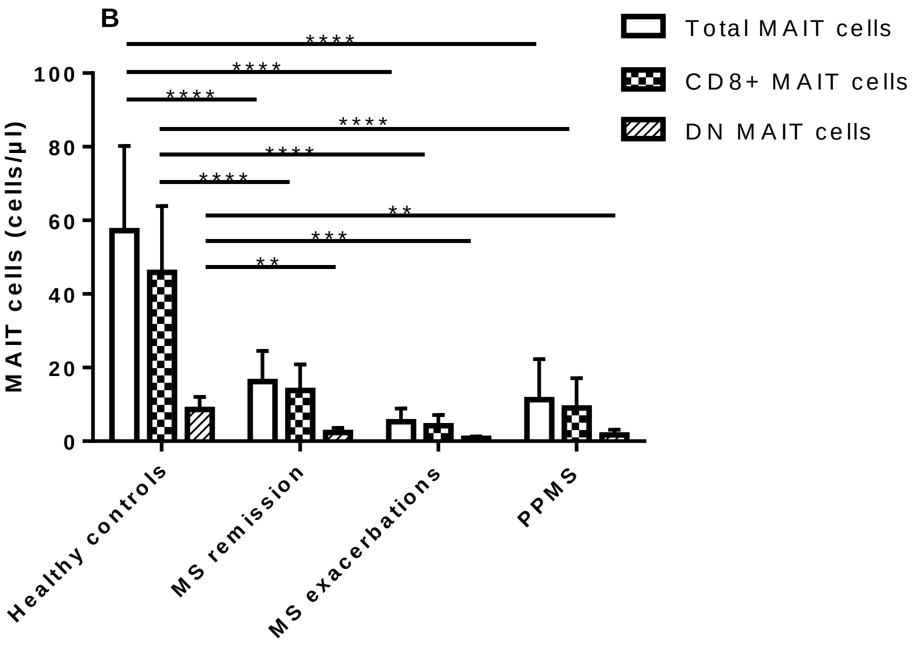

Data are presented as mean values ± SEM from seven different experiments. Statistical analysis was performed using the Wilcoxon matched-pairs signed rank test. $**p < 0.01$, $***p < 0.001$, $****p < 0.0001$.
