# Supplementary figures and images for: Mucosal-Associated Invariant T Cell Features and TCR Repertoire Characteristics During the Course of Multiple Sclerosis
Source: Front Immunol. 2019 Nov 20;10:2690. doi: 10.3389/fimmu.2019.02690 (PMC6880779; doi:10.3389/fimmu.2019.02690)

Supplementary figure S2

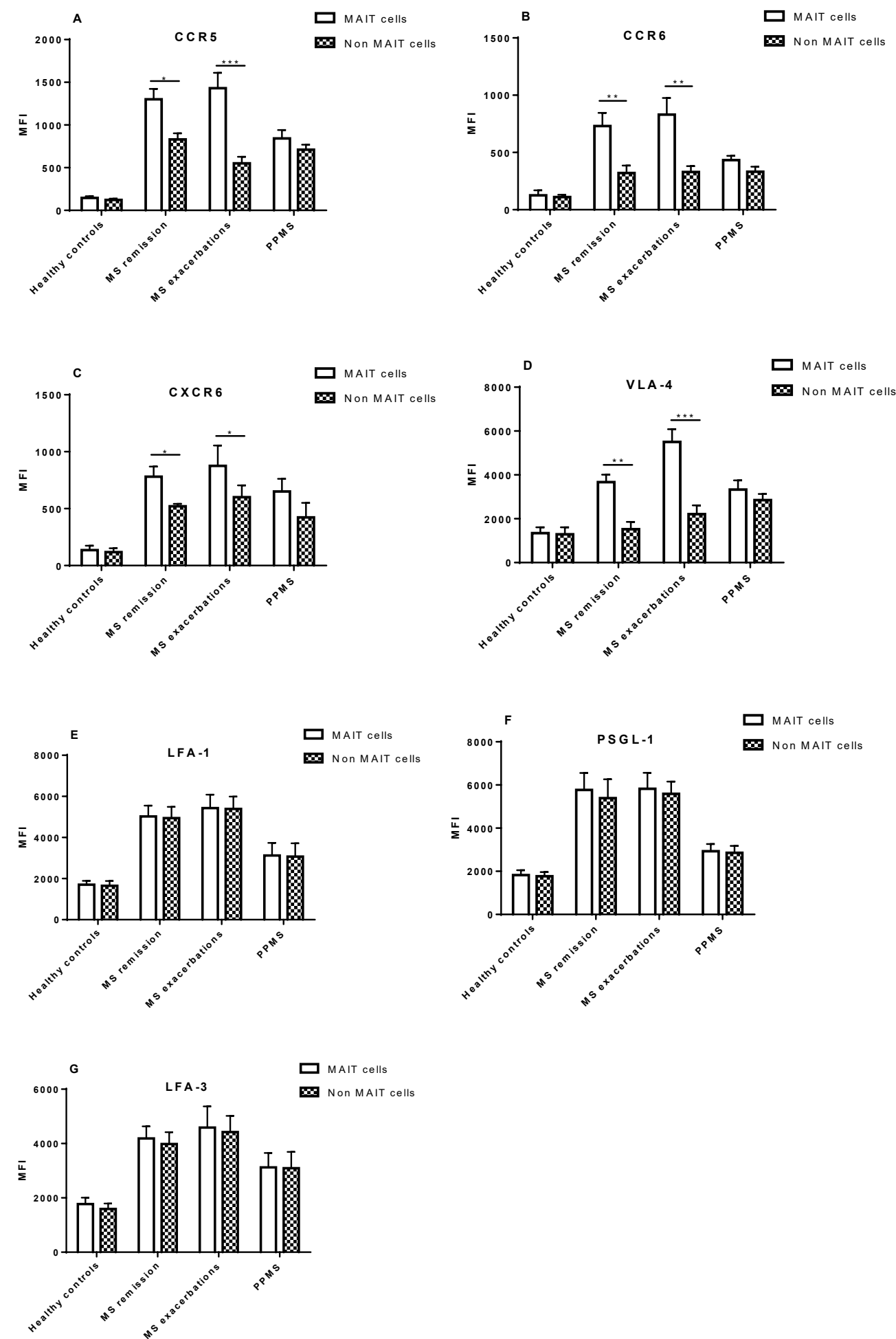

Supplement: Supplementary Figure S2 — (A–C) Expression of CCR5, CCR6, and CXCR6 in both MAIT and non-MAIT cells. (D–G) Expression of integrins and adhesion molecules VLA-4, LFA-1, PSGL-1, and LFA-3 in both MAIT and non-MAIT cells. In all cases data are presented as mean values ± SEM from 24 RRMS patients (MS in remission = 12 and MS during exacerbations = 12), 12 PPMS patients and 12 healthy controls. Statistical analysis was performed using the Wilcoxon matched-pairs signed rank test (A–D). *p < 0.05, **p < 0.01, ***p < 0.001. [file Image_2.pdf]
