# Supplementary material for: Mucosal-Associated Invariant T Cell Features and TCR Repertoire Characteristics During the Course of Multiple Sclerosis
Source: Front Immunol. 2019 Nov 20;10:2690. doi: 10.3389/fimmu.2019.02690 (PMC6880779; doi:10.3389/fimmu.2019.02690)
Supplement: Supplementary Figure S3 — Correlations between MAIT cell numbers and MRI lesions estimated using Spearman's correlation. (A) New Gd+ lesions. (B) New or enlarging T2 lesions. (C) CUA score. [file Image_3.pdf]

Supplementary figure S3. Correlations between MAIT cell numbers and MRI lesions estimated using Spearman‘s correlation.

A New Gd+ lesions

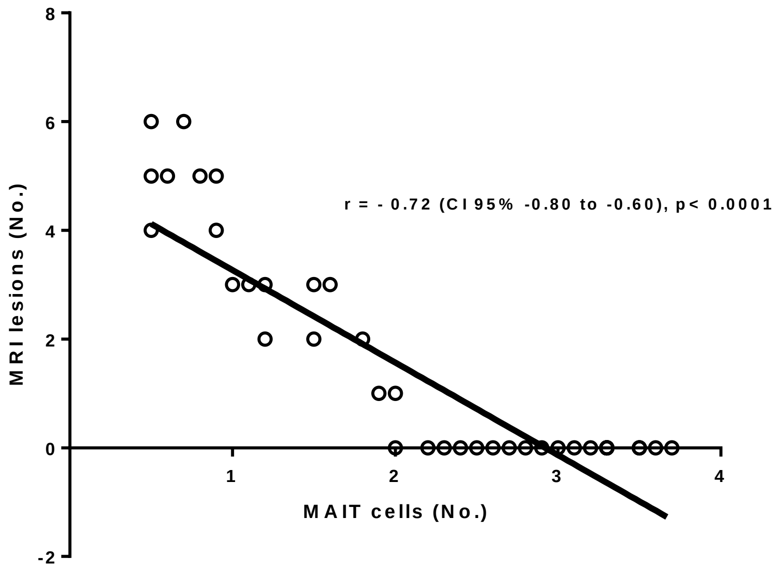

B New or enlarging T2 lesions

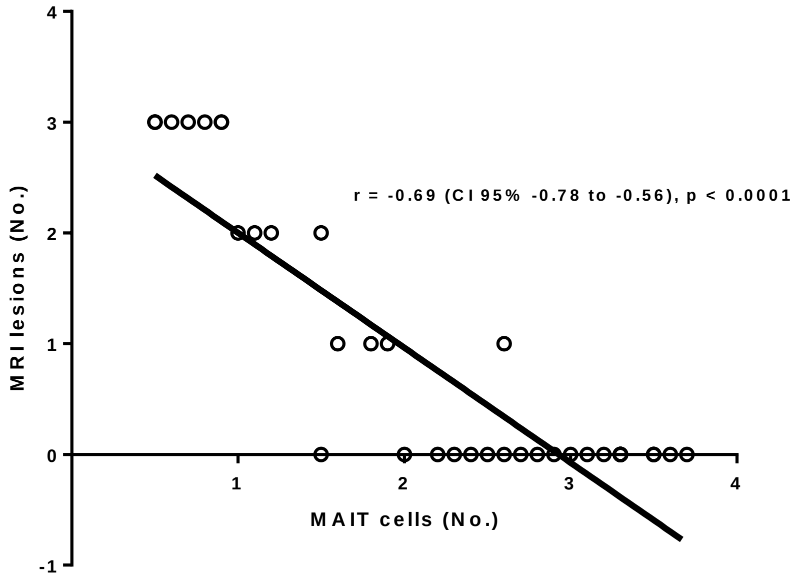

C CUA score

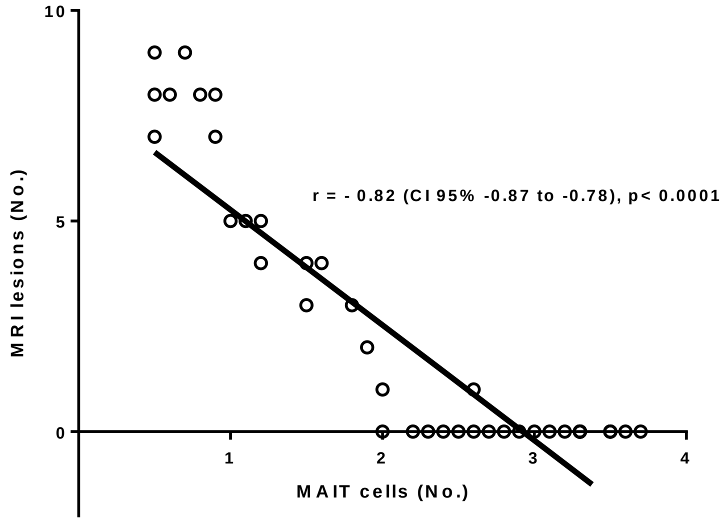

.
