# Supplementary material for: Mucosal-Associated Invariant T Cell Features and TCR Repertoire Characteristics During the Course of Multiple Sclerosis
Source: Front Immunol. 2019 Nov 20;10:2690. doi: 10.3389/fimmu.2019.02690 (PMC6880779; doi:10.3389/fimmu.2019.02690)
Supplement: S1 Table — Phenotype and TCRβ amino acid sequences of MAIT cell clones isolated from blood of RRMS patients at the beginning of the study. [file Table_1.docx]

**S1 Table . Phenotype and TCRβ amino acid sequences of clones of MAIT cells isolated from the blood of RRMS patients at the beginning of the study**

| **Patient** | **Clone** | **CD4** | **CD8** | **CD161** | **CDR3 AA sequence** | **TRBV^a^** | **TRBJ** |
| --- | --- | --- | --- | --- | --- | --- | --- |
| **1** | 1-1-PB | - | - | + | ASRLMSGSSYEQY | 6-1 | 01-05 |
|  | 1-2-PB | - | + | + | ASSSSGGDTQY | 6-4 | 02-01 |
|  | 1-3-PB | - | + | + | ASRLMSGSSYEQY | 6-1 | 01-05 |
|  | 1-4-PB | - | + | + | SARDRRETEAF | 20-1 | 02-07 |
|  | 1-5-PB | - | + | + | CASSSGSTSYNEQ | 7-6 | 02-01 |
|  | 1-6-PB | - | + | + | CASSQDRGSQPQH | 6-5 | 01-05 |
|  | 1-7-PB | - | + | + | SARDRRETEAF | 20-1 | 02-07 |
| **2** | 2-1-PB | - | - | + | ASRLMSGSSYEQY | 6-1 | 02-01 |
|  | 2-2-PB | - | + | + | ASRLMSGSSYEQY | 6-1 | 01-02 |
|  | 2-3-PB | - | + | + | ASSSSGGDTQY | 6-4 | 02-01 |
| **3** | 3-1-PB | - | + | + | ASSLGSSGNTIY | 14 | 02-07 |
|  | 3-2-PB | - | - | + | ASSSSGGDTQY | 6-4 | 02-01 |
|  | 3-3-PB | - | + | + | CASSQDRGSQPQH | 6-5 | 01-05 |
| **4** | 4-1-PB | - | + | + | ASSLGSSGNTIY | 14 | 02-07 |
|  | 4-2-PB | - | + | + | ASRLMSGSSYEQY | 6-1 | 01-05 |
|  | 4-3-PB | - | - | + | SARDRRETEAF | 20-1 | 01-02 |
|  | 4-4-PB | - | + | + | CASSDSSRGVPYEQFF | 6-4 | 02-01 |
|  | 4-5-PB | - | + | + | CASSQDRGSQPQH | 6-5 | 01-05 |
| **5** | 5-1-PB | - | - | + | SARGDREAYNEQF | 20-1 | 01-02 |
|  | 5-2-PB | - | + | + | ASRLMSGSSYEQY | 6-1 | 01-05 |
|  | 5-3-PB | - | + | + | SARGDREAYNEQF | 20-1 | 01-02 |
|  | 5-4-PB | - | - | + | CASSYGGVGQPQH | 7-9 | 01-03 |
|  | 5-5-PB | - | + | + | ASRLMSGSSYEQY | 6-1 | 02-01 |
|  | 5-6-PB | - | + | + | SARGDREAYNEQF | 20-1 | 02-07 |
| **6** | 6-1-PB | - | + | + | CASSDYGAGHNEQF | 6-4 | 02-01 |
|  | 6-2-PB | - | + | + | SARGDREAYNEQF | 20-1 | 01-02 |
|  | 6-3-PB | - | - | + | SARDRRETEAF | 20-1 | 01-02 |
|  | 6-4-PB | - | + | + | ASRLMSGSSYEQY | 6-1 | 01-02 |
|  | 6-5-PB | - | + | + | CASSSGSTSYNEQ | 7-6 | 02-01 |
|  | 6-6-PB | - | + | + | SARDRRETEAF | 20-1 | 01-02 |
|  | 6-7-PB | - | - | + | ASSLGSSGNTIY | 14 | 02-07 |
| **7** | 7-1-PB | - | + | + | SARGDREAYNEQF | 20-1 | 02-07 |
|  | 7-2-PB | - | + | + | CASSSGSTSYNEQ | 7-6 | 02-01 |
|  | 7-3-PB | - | - | + | SARGDREAYNEQF | 20-1 | 02-07 |

^a^According to IMGT nomenclature
