# Supplementary material for: Mucosal-Associated Invariant T Cell Features and TCR Repertoire Characteristics During the Course of Multiple Sclerosis
Source: Front Immunol. 2019 Nov 20;10:2690. doi: 10.3389/fimmu.2019.02690 (PMC6880779; doi:10.3389/fimmu.2019.02690)
Supplement: S2 Table — Phenotype and TCRβ amino acid sequences of MAIT cell clones isolated from CSF of RRMS patients. [file Table_2.docx]

**S2 Table. Phenotype and TCRβ amino acid sequences of MAIT cell clones isolated from CSF of RRMS patients**

| **Patient** | **Clone** | **CD4** | **CD8** | **CD161** | **CD103** | **Cytokine** | **CDR3 AA sequence** | **TRBV^a^** | **TRBJ** |
| --- | --- | --- | --- | --- | --- | --- | --- | --- | --- |
| **1** | 1-1-CSF | - | - | + | + | IL-17 | ASSSSGGDTQY | 6-4 | 02-01 |
|  | 1-2-CSF | - | - | + | + | IL-17 | SARDRRETEAF | 20-1 | 02-07 |
|  | 1-3-CSF | - | + | + | + | IFN-γ | ASRLMSGSSYEQY | 6-1 | 01-05 |
|  | 1-4-CSF | - | - | + | + | IFN-γ | ASSLGSSGNTIY | 14 | 02-07 |
|  | 1-5-CSF | - | + | + | - | GM-CSF | SARDRRETEAF | 20-1 | 02-07 |
|  | 1-6-CSF | - | + | + | + | IL-17 | ASRLMSGSSYEQY | 6-1 | 01-05 |
| **4** | 4-1-CSF | - | + | + | + | IFN-γ | CASSDSSRGVPYEQFF | 6-4 | 02-01 |
|  | 4-2-CSF | - | + | + | + | IFN-γ | SARDRRETEAF | 20.1 | 02-07 |
|  | 4-3-CSF | - | - | + | + | GM-CSF | ASRLMSGSSYEQY | 6-1 | 01-05 |
|  | 4-4-CSF | - | + | + | + | IL-17 | ASSLGSSGNTIY | 14 | 02-07 |
| **5** | 5-1-CSF | - | - | + | - | IFN-γ | SARGDREAYNEQF | 20-1 | 02-07 |
|  | 5-2-CSF | - | + | + | + | IL-17 | ASSLGSSGNTIY | 14 | 02-07 |
|  | 5-3-CSF | - | + | + | - | IFN-γ | ASRLMSGSSYEQY | 6-1 | 01-05 |
|  | 5-4-CSF | - | - | + | + | IL-17 | SARGDREAYNEQF | 20-1 | 01-02 |
|  | 5-5-CSF | - | - | + | + | GM-CSF | CASSQDRGSQPQH | 6-5 | 01-05 |
|  | 5-6-CSF | - | + | + | + | IL-17 | SARGDREAYNEQF | 20-1 | 01-02 |
|  | 5-7-CSF |  |  |  | + | IFN-γ | ASRLMSGSSYEQY | 6-1 | 02-01 |
| **6** | 6-1-CSF | - | - | + | + | IL-17 | SARDRRETEAF | 20-1 | 01-02 |
|  | 6-2-CSF | - | + | + | + | IFN-γ | CASSSGSTSYNEQ | 7-6 | 02-01 |
|  | 6-3-CSF | - | + | + | - | IFN-γ | CASSSGSTSYNEQ | 7-6 | 02-01 |
|  | 6-4-CSF | - | + | + | + | GM-CSF | SARGDREAYNEQF | 20-1 | 01-02 |
|  | 6-5-CSF | - | + | + | + | IFN-γ | SARGDREAYNEQF | 20-1 | 02-07 |
|  | 6-6-CSF | - | - | + | + | IL-17 | SARDRRETEAF | 20-1 | 01-02 |

^a^According to IMGT nomenclature
