# Supplementary material for: Mucosal-Associated Invariant T Cell Features and TCR Repertoire Characteristics During the Course of Multiple Sclerosis
Source: Front Immunol. 2019 Nov 20;10:2690. doi: 10.3389/fimmu.2019.02690 (PMC6880779; doi:10.3389/fimmu.2019.02690)
Supplement: S3 Table — Phenotype and TCRβ amino acid sequences of MAIT cell clones isolated from peripheral blood and CSF from healthy controls. [file Table_3.docx]

**S3 Table. Phenotype and TCRβ amino acid sequences of MAIT cell clones isolated from peripheral blood and CSF from healthy controls**

| **Control** | **Clone** | **CD4** | **CD8** | **CD161** | **Cytokine** | **CDR3 AA sequence** | **TRBV^a^** | **TRBJ** |
| --- | --- | --- | --- | --- | --- | --- | --- | --- |
| **Peripheral blood** |  |  |  |  |  |  |  |  |
| **1** | HC1-1-PB | - | + | + | IL-17 | SARDRRETEAF | 20-1 | 02-07 |
|  | HC1-2-PB | - | + | + | IL-17 | ASRLMSGSSYEQY | 6-1 | 01-02 |
|  | HC1-3-PB | - | + | + | ND | ASSSSGGDTQY | 6-4 | 02-01 |
|  | HC1-4-PB | - | + | + | ND | ASRLMSGSSYEQY | 6-1 | 01-05 |
|  | HC1-5-PB | - | + | + | ND | SARDRRETEAF | 20-1 | 01-02 |
| **2** | HC2-1-PB | - | + | + | IFN-γ | ASSLGSSGNTIY | 20-1 | 01-02 |
|  | HC2-2-PB | - | + | + | ND | ASSQERGSQETQY | 4-3 | 01-03 |
|  | HC2-3-PB | - | + | + | ND | SARDRRETEAF | 20-1 | 01-02 |
|  | HC2-4-PB | - | - | + | IL-17 | ASSLGSSGNTIY | 14 | 02-07 |
|  | HC2-5-PB | - | - | + | IFN-γ | CASSQDRGSQPQH | 6-5 | 01-05 |
|  | HC2-6-PB | - | + | + | ND | ASSLGSSGNTIY | 14 | 02-07 |
| **3** | HC3-1-PB | - | + | + | GM-CSF | SARDRRETEAF | 20-1 | 01-02 |
|  | HC3-2-PB | - | + | + | IL-17 | SARDRRETEAF | 20-1 | 01-02 |
|  | HC3-3-PB | - | + | + | ND | ASSQERGSQETQY | 4-3 | 01-03 |
|  | HC3-4-PB | - | + | + | ND | ASSLGSSGNTIY | 14 | 02-07 |
|  | HC3-5-PB | - | + | + | ND | CASSQDRGSQPQH | 6-5 | 01-05 |
| **CSF** |  |  |  |  |  |  |  |  |
| **1** | HC1-1-CSF | - | - | + | ND | ASSLGSSGNTIY | 14 | 02-07 |
|  | HC1-2-CSF | - | - | + | ND | CASSQDRGSQPQH | 6-5 | 01-05 |
| **2** | HC2-1-CSF | - | + | + | ND | ASSLGSSGNTIY | 4-3 | 01-03 |
|  | HC2-2-CSF | - | + | + | IL-17 | ASRLMSGSSYEQY | 6-1 | 01-02 |
| **3** | HC3-1-CSF | - | + | + | IFN-γ | ASSSSGGDTQY | 6-4 | 02-01 |
|  | HC3-2-CSF | - | + | + | ND | ASRLMSGSSYEQY | 6-1 | 01-05 |
|  | HC3-3-CSF | - | + | + | ND | ASRLMSGSSYEQY | 6-1 | 01-02 |

^a^According to IMGT nomenclature; ND: not detected
