# Supplementary material for: Mucosal-Associated Invariant T Cell Features and TCR Repertoire Characteristics During the Course of Multiple Sclerosis
Source: Front Immunol. 2019 Nov 20;10:2690. doi: 10.3389/fimmu.2019.02690 (PMC6880779; doi:10.3389/fimmu.2019.02690)
Supplement: S4 Table — Longitudinal analysis of the TCRβ chain sequence frequency in clones of MAIT cells isolated from patients with RRMS over time. [file Table_4.docx]

**S4 Table. Longitudinal analysis of the sequence frequency of the TCRβ chain in clones of MAIT cells isolated from patients with RRMS over time**

**Patient AA sequence TRBV^b^ TRBJ Percentage present in MAIT cells by year (%)**

|  |  |  |  | **Study entry** | **Year 1** | **Year 2** | **Year 3** |
| --- | --- | --- | --- | --- | --- | --- | --- |
| **1** |  |  |  | **n=7^c^** | **n=12** | **n=20** | **n=23** |
|  | ***ASRLMSGSSYEQY^d^*** | 6-1 | 01-05 | 28.5 | 25 | 15 | 21,7 |
|  | ***ASSSSGGDTQY*** | 6-4 | 02-01 | 14.3 | 25 | 15 | 13,0 |
|  | ***SARDRRETEAF*** | 20-1 | 02-07 | 28.5 | 16.6 | 20 | 17,3 |
|  | ***CASSSGSTSYNEQ*** | 7-6 | 02-01 | 14.3 | 8.3 | 10 | 17,3 |
|  | ***CASSQDRGSQPQH*** | 6-5 | 01-05 | 14.3 | 16.6 | 15 | 13,0 |
|  | CASSLASNGYTF | 7-9 | 01-02 | 0 | 0 | 10 | 0 |
|  | CASATWAGATDTQYF | 19 | 02-03 | 0 | 0 | 5 | 4,3 |
|  | CASSPSSSGPYEQYF | 18 | 02-07 | 0 | 0 | 0 | 4,3 |
|  | CAGGTGSDTQYF | 5-4 | 02-03 | 0 | 0 | 5 | 4,3 |
|  | ASSLGSSGNTIY | 14 | 02-07 | 0 | 8,3 | 5 | 4,3 |
| **2** |  |  |  | **n =3** | **n=10** | **n=12** | **n=15** |
|  | ***ASRLMSGSSYEQY*** | 6-1 | 02-01 | 33.3 | 30 | 25 | 20 |
|  | ***ASRLMSGSSYEQY*** | 6-1 | 01-02 | 33.3 | 30 | 25 | 20 |
|  | ***ASSSSGGDTQY*** | 6-4 | 02-01 | 33.3 | 30 | 16.7 | 13.3 |
|  | SARGDREAYNEQF | 20-1 | 02-07 | 0 | 10 | 16.7 | 20 |
|  | CASSDYGAGHNEQF | 6-4 | 02-01 | 0 | 0 | 8.3 | 13.3 |
|  | CASSYGGVGQPQH | 7-9 | 01-03 | 0 | 0 | 8.3 | 13.3 |
|  |  |  |  |  |  |  |  |
| **3** |  |  |  | **n=3** | **n=9** | **n=10** | **n=13** |
|  | ***ASSLGSSGNTIY*** | 14 | 02-07 | 33.3 | 33.3 | 20 | 15.4 |
|  | ***ASSSSGGDTQY*** | 6-4 | 02-01 | 33.3 | 33.3 | 30 | 23 |
|  | ***CASSQDRGSQPQH*** | 6-5 | 01-05 | 33.3 | 33.3 | 30 | 15.4 |
|  | SARGDREAYNEQF | 20-1 | 02-07 | 0 | 0 | 10 | 7.7 |
|  | ASRLMSGSSYEQY | 6-1 | 01-02 | 0 | 0 | 0 | 15.4 |
|  | ASRLMSGSSYEQY | 6-1 | 01-05 | 0 | 0 | 0 | 15.4 |
|  | CASSSGSTSYNEQ | 7-6 | 02-01 | 0 | 0 | 10 | 7.7 |
| **4** |  |  |  | **n =5** | **n=15** | **n=18** | **n=25** |
|  | ***ASSLGSSGNTIY*** | 14 | 02-07 | 20 | 20 | 22.2 | 20 |
|  | ***ASRLMSGSSYEQY*** | 6-1 | 01-05 | 20 | 6,6 | 11.1 | 12 |
|  | ***SARDRRETEAF*** | 20-1 | 01-02 | 20 | 20 | 22.2 | 20 |
|  | ***CASSDSSRGVPYEQFF*** | 6-4 | 02-01 | 20 | 20 | 16.6 | 16 |
|  | ***CASSQDRGSQPQH*** | 6-5 | 01-05 | 20 | 6,6 | 11.1 | 12 |
|  | CASSYGGVGQPQH | 7-9 | 01-03 | 0 | 13.3 | 11.1 | 4 |
|  | CASSSGSTSYNEQ | 7-6 | 02-01 | 0 | 6.6 | 5.5 | 4 |
|  | ASRLMSGSSYEQY | 6-1 | 01-02 | 0 | 6.6 | 0 | 8 |
|  | SARDRRETEAF | 20-1 | 02-07 | 0 | 0 | 0 | 4 |
| **5** |  |  |  | **n =6** | **n=15** | **n=22** | **n=28** |
|  | ***SARGDREAYNEQF*** | 20-1 | 01-02 | 33.3 | 26.6 | 22.7 | 21.4 |
|  | ***ASRLMSGSSYEQY*** | 6-1 | 01-05 | 16.6 | 20 | 18.2 | 21.4 |
|  | ***CASSYGGVGQPQH*** | 7-9 | 01-03 | 16.6 | 13.3 | 13.6 | 14.3 |
|  | ***ASRLMSGSSYEQY*** | 6-1 | 02-01 | 16.6 | 20 | 18.2 | 14.3 |
|  | ***SARGDREAYNEQF*** | 20-1 | 02-07 | 16.6 | 13.3 | 13.6 | 14.3 |
|  | ASSLGSSGNTIY | 14 | 02-07 | 0 | 6.6 | 9 | 7.1 |
|  | CASSQDRGSQPQH | 6-5 | 01-05 | 0 | 0 | 4.5 | 7.1 |
| **6** |  |  |  | **n =7** | **n=20** | **n=18** | **n=22** |
|  | ***CASSDYGAGHNEQF*** | 6-4 | 02-01 | 28.5 | 20 | 22.2 | 18.2 |
|  | ***SARGDREAYNEQF*** | 20-1 | 01-02 | 14.3 | 10 | 11.1 | 13.6 |
|  | ***SARDRRETEAF*** | 20-1 | 01-02 | 14.3 | 15 | 16.6 | 18.2 |
|  | ***ASRLMSGSSYEQY*** | 6-1 | 01-02 | 14.3 | 10 | 5.5 | 9.1 |
|  | ***CASSSGSTSYNEQ*** | 7-6 | 02-01 | 14.3 | 15 | 16.6 | 9.1 |
|  | ***ASSLGSSGNTIY*** | 14 | 02-07 | 14.3 | 10 | 11.1 | 9.1 |
|  | CASSYGGVGQPQH | 7-9 | 01-03 | 0 | 5 | 5.6 | 13.6 |
|  | CASSQDRGSQPQH | 6-5 | 01-05 | 0 | 5 | 5.6 | 0 |
|  | ASRLMSGSSYEQY | 6-1 | 01-05 | 0 | 0 | 5.6 | 9.1 |
| **7** |  |  |  | **n =3** | **n=15** | **n=17** | **n=28** |
|  | ***SARGDREAYNEQF*** | 20-1 | 02-07 | 66.6 | 33.3 | 29.4 | 28.6 |
|  | ***CASSSGSTSYNEQ*** | 7-6 | 02-01 | 33.3 | 20 | 23.5 | 25 |
|  | SARDRRETEAF | 20-1 | 01-02 | 0 | 13.3 | 17.6 | 14.3 |
|  | ASSLGSSGNTIY | 14 | 02-07 | 0 | 6.5 | 0 | 7.1 |
|  | ASRLMSGSSYEQY | 6-1 | 01-05 | 0 | 13.3 | 11.8 | 10.7 |
|  | CASSQDRGSQPQH | 6-5 | 01-05 | 0 | 13.6 | 17.6 | 14.3 |

^a^Values represent frequency of AA sequences relative to the total number of MAIT cell clones isolated at each time point; ^b^According to **International ImMunoGeneTics information system® nomenclature; ^c^ Number of MAIT cell clones isolated at each time point. In some cases more than one clone expresses the same TRBV and TRBJ;** ^d^Bold and italics indicate the AA sequences corresponding to CDR3β clonotypes identified in 7 MAIT cell clones isolated at the beginning of the study (time 0).
